# A Heterogeneous Manganese Catalyst for the Selective Hydrogenation of Nitroarenes

**DOI:** 10.1021/jacs.5c19788

**Published:** 2026-03-26

**Authors:** Jianglin Duan, Wu Li, Yujing Ren, Kathrin Junge, Matthias Beller

**Affiliations:** † Leibniz-Institut für Katalyse, Rostock 18059, Germany; ‡ Interdisciplinary Research Center of Biology and Catalysis, School of Life Science and Technology, 26487 Northwestern Polytechnical University, Xi’an 710072, China

## Abstract

Catalytic hydrogenations of nitroarenes are fundamental processes in organic chemistry, allowing for atom-efficient and clean synthesis of valuable amines. Recently, earth-abundant metals have attracted considerable interest in this field. Surprisingly, despite more than a century of developments in heterogeneous catalysis, in the present study, we describe for the first time a specific manganese-based single-atom catalyst that allows the selective hydrogenation of various nitroarenes. The corresponding amines were obtained with a high selectivity, especially for nitrostyrene. In the hydrogenation process, the addition of H_2_O was found to promote the activity of Mn_1_–N-C/Al_2_O_3_. Mechanistic control experiments indicated that the heterolytic activation of hydrogen may be a contributing factor. This work provides an effective approach for the design of completely new Mn-based heterogeneous hydrogenation catalysts.

## Graphical abstract

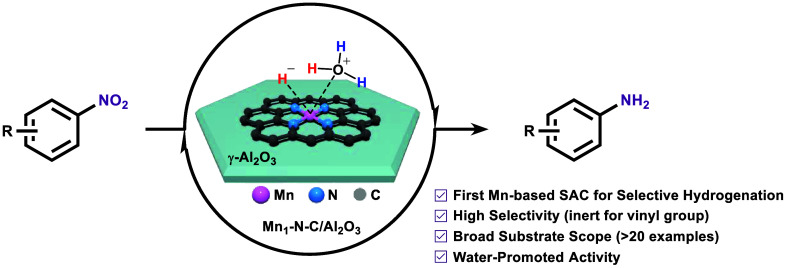

## Introduction

In modern industrial development, catalysis serves as a fundamental cornerstone in the chemical and energy sectors. As the core of catalysis, the catalyst plays a crucial role in increasing reaction rate, controlling selectivity, and reducing energy consumption. Today, approximately 90% of industrial chemical transformations worldwide rely on catalytic processes, highlighting the application of catalysts as a pivotal force in modern chemical engineering, energy, environmental protection, pharmaceuticals, and fine chemicals.^1–9^ Among all catalysts, platinum group metal (PGM) catalysts often exhibit outstanding catalytic performance and are widely used in many important catalytic processes.^10–14^ For example, heterogeneous Pt-based catalysts have attracted much attention in the steam reforming reaction (e.g., Pt/α-MoC),^15^ while molecular Pd-based catalysts are widely used in cross-coupling reactions.^16^ However, the limited availability and cost of precious metals restrict their practical application. In this context, the development of nonprecious metal materials with high efficiency, low cost, and robust properties in industrial catalytic processes continues to be of great importance.

Comparing the many transformations of PGM catalysts, hydrogenation reactions stand out as one of the most versatile tools in the petrochemical, fine chemical, and environmental industries. In fact, it is estimated that approximately 25% of chemical processes involve at least one hydrogenation step.^17–19^ Among these transformations, the hydrogenation of nitroarenes to anilines is a well-established process in both industry and academic laboratories. Industrially, this reaction is typically achieved using commercial noble-metal catalysts (e.g., Lindlar and NanoSelect catalysts), enabling the production of anilines that serve as key intermediates for pharmaceuticals, agrochemicals, dyes, and polymers. Academically, the nitroarene hydrogenation reaction has been extensively investigated since its first report in 1842,^20^ evolving into a model transformation for studying reaction mechanisms, chemoselectivity control, and catalyst design. Despite its apparent maturity, this classical reduction continues to attract significant scientific and economic interest, particularly driven by the urgent need to replace traditional PGM-based catalysts with earth-abundant, non-noble-metal alternatives.^21–26^ For the synthesis of advanced amine building blocks, which are valuable intermediates in modern synthesis, achieving high chemoselectivity in such reactions is crucial. Apart from supported Au nanoparticles, notable examples of iron- ^27–29^ and cobalt-based^30–33^ heterogeneous catalysts for general and selective hydrogenation of nitroarenes to amines have been disclosed.

Compared with other active catalyst metals, manganese species are relatively nontoxic, and Mn is the third most abundant transition metal in the Earth’s crust, after Fe and Ti.^34–36^ Despite these advantages, Mn remains underutilized in heterogeneous catalysis. Although Kida prepared a Mn/graphene catalyst for hydrogenolysis of the C–OH bond in hydroxymethylfurfural,^37^ to the best of our knowledge, no heterogeneous catalysts with active manganese centers have been reported for the selective hydrogenation of nitroarenes. Interestingly, in the past decade, molecularly defined Mn complexes have emerged as a new class of hydrogenation catalysts, and this field became a hot topic in contemporary homogeneous catalysis (Figure 1A).^38–46^

**Figure 1:**
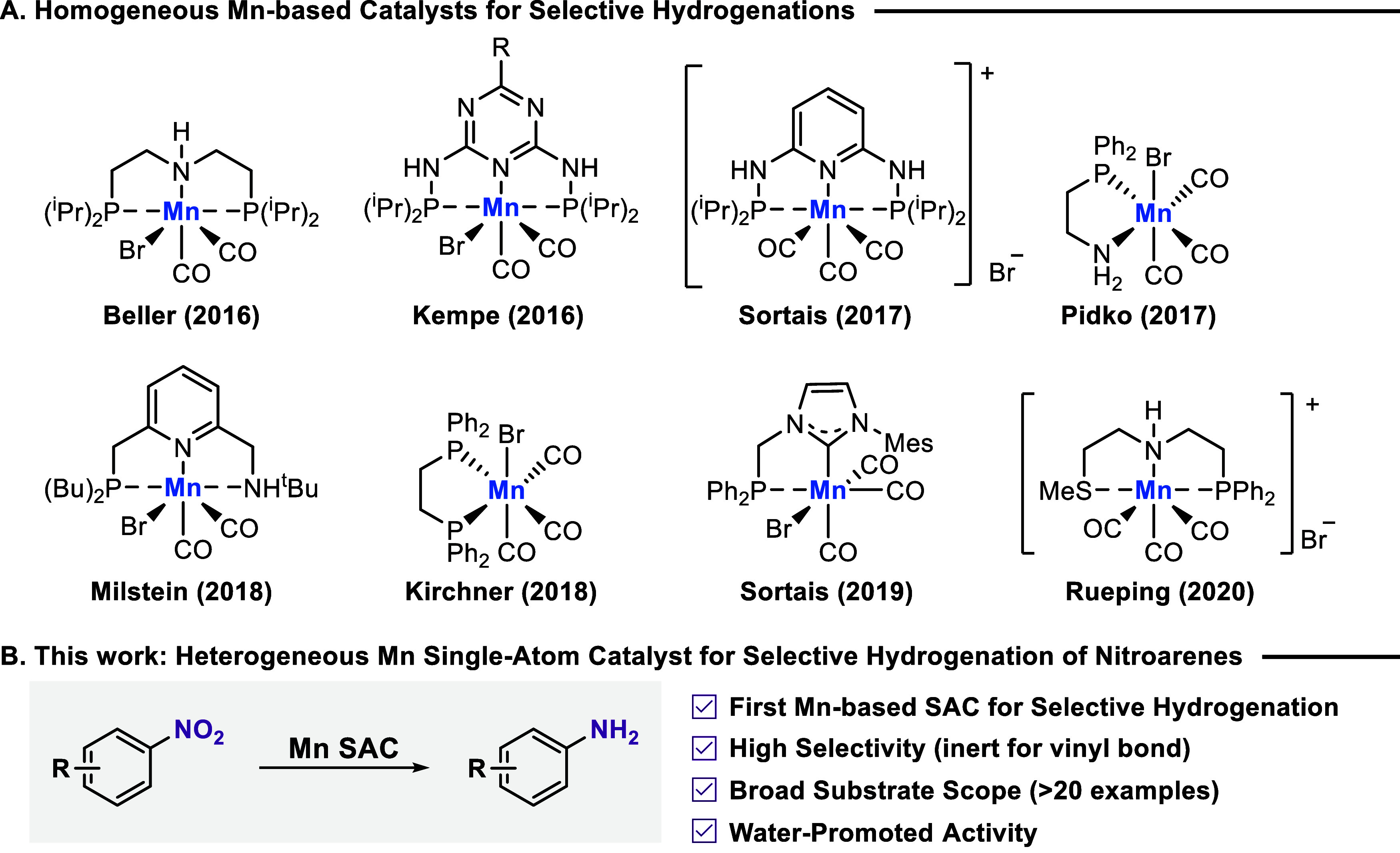
Selected examples of active Mn-based hydrogenation catalysts. (A) Homogeneous Mn-based catalysts for selective hydrogenations (e.g., nitriles, ketones, aldehydes, esters, and alkynes). (B) Heterogeneous Mn single-atom catalyst for selective hydrogenation of nitroarenes.

Although most supported manganese particles do not allow catalytic hydrogenation due to their intrinsic inertness to hydrogen activation, the success of homogeneous Mn complexes in hydrogenation makes it likely that it is possible. Meanwhile, recent studies on Mn–N–C materials in electrocatalytic fields have demonstrated that well-defined Mn–N_*x*_ coordination environments can effectively stabilize atomically dispersed Mn centers.^47^ In this context, we had the idea to use single-atom Mn catalysts (Mn-SACs).^48–50^ SACs have recently emerged as a platform for bridging different subfields of catalysis (Das, Waiba et al.).^51^ Due to the fact that all active metal atoms are isolated on the support, SACs not only allow for maximum efficiency in atom utilization compared with conventional heterogeneous counterparts but also resemble the catalytic properties of homogeneous metal complexes.^52^

Inspired by this and based on our previous work on homogeneous Mn catalysis,^53,54^ we present here the first Mn-SAC (Mn_1_-N-C/Al_2_O_3_) for selective hydrogenation reactions (Figure 1B). In the optimal catalyst material, defined Mn_1_-N_4_ single-atom sites were successfully anchored on the nitrogen-doped carbon surface after pyrolysis. Mechanistic studies reveal that the presence of H_2_O promotes heterolytic cleavage of H_2_ and the overall hydrogenation reaction. As a result, a wide range of substrates and high selectivity can be achieved in the hydrogenation of nitroarenes.

## Results and Discussion

### Preparation and Characterization of Mn_1_-N-C/Al_2_O_3_

At the start of this project, three different Mn­(II) salts (Mn­(OAc)_2_·4H_2_O, MnCl_2_·4H_2_O, and Mn­(NO_3_)_2_·4H_2_O) were mixed with three nitrogen-containing ligands (melamine, 1,10-phenanthroline, and phenylalanine). The latter were selected based on availability, nitrogen content, and previous use in pyrolysis processes with other metals.^27,55,56^ After physical immobilization of both components on inert carbon or metal oxides, e.g., γ-Al_2_O_3_, TiO_2_, and nanodiamond (ND), the resulting solids were pyrolyzed at 400–800 °C (Table S1). It is worth mentioning that the introduction of γ-Al_2_O_3_ or TiO_2_ is beneficial for the dispersion of Mn species during the pyrolysis process.^57^ The general procedure for the most active material, Mn_1_-N-C/Al_2_O_3_, is illustrated in the Supporting Information. From the N_2_ adsorption–desorption result (Figure S1), the *S*_BET_ of Mn_1_-N-C/Al_2_O_3_ is ∼135 m^2^/g (Table S2). Inductively coupled plasma (ICP) analysis showed that the Mn loading in Mn_1_-N-C/Al_2_O_3_ is 2.86 wt % (Table S3). According to the total element analysis result, no other known hydrogenation metal, including Ru, Rh, Pd, Ir, Pt, Fe, Co, Ni, and Cu, was detected in Mn_1_-N-C/Al_2_O_3_.

The X-ray diffraction (XRD) pattern of Mn_1_-N-C/Al_2_O_3_ displays a characteristic peak of γ-Al_2_O_3_ (Figure 2A), in the absence of any metallic Mn or Mn oxide phase. Also, no noticeable presence of Mn species was detected in the scanning electron microscopy (SEM) images (Figure S2). Consistently, the scanning transmission electron microscopy (STEM) and corresponding energy dispersive spectrometer (EDS) analysis of Mn_1_-N-C/Al_2_O_3_ reveal a clear and uniform distribution of C, N, O, Al, and Mn species on the γ-Al_2_O_3_ support (Figures 2B and S3). These structural characterization results suggest a high dispersion of the Mn species. To investigate the dispersion of Mn, aberration-corrected HAADF-STEM (AC-HAADF-STEM) characterization was performed. As shown in Figures 2C and S4, isolated and easily distinguishable bright spots were observed, which are attributed to Mn_1_ single atoms. Summarizing various analytical methods, the successful synthesis of Mn_1_-N-C/Al_2_O_3_ SAC can be assumed.

**Figure 2:**
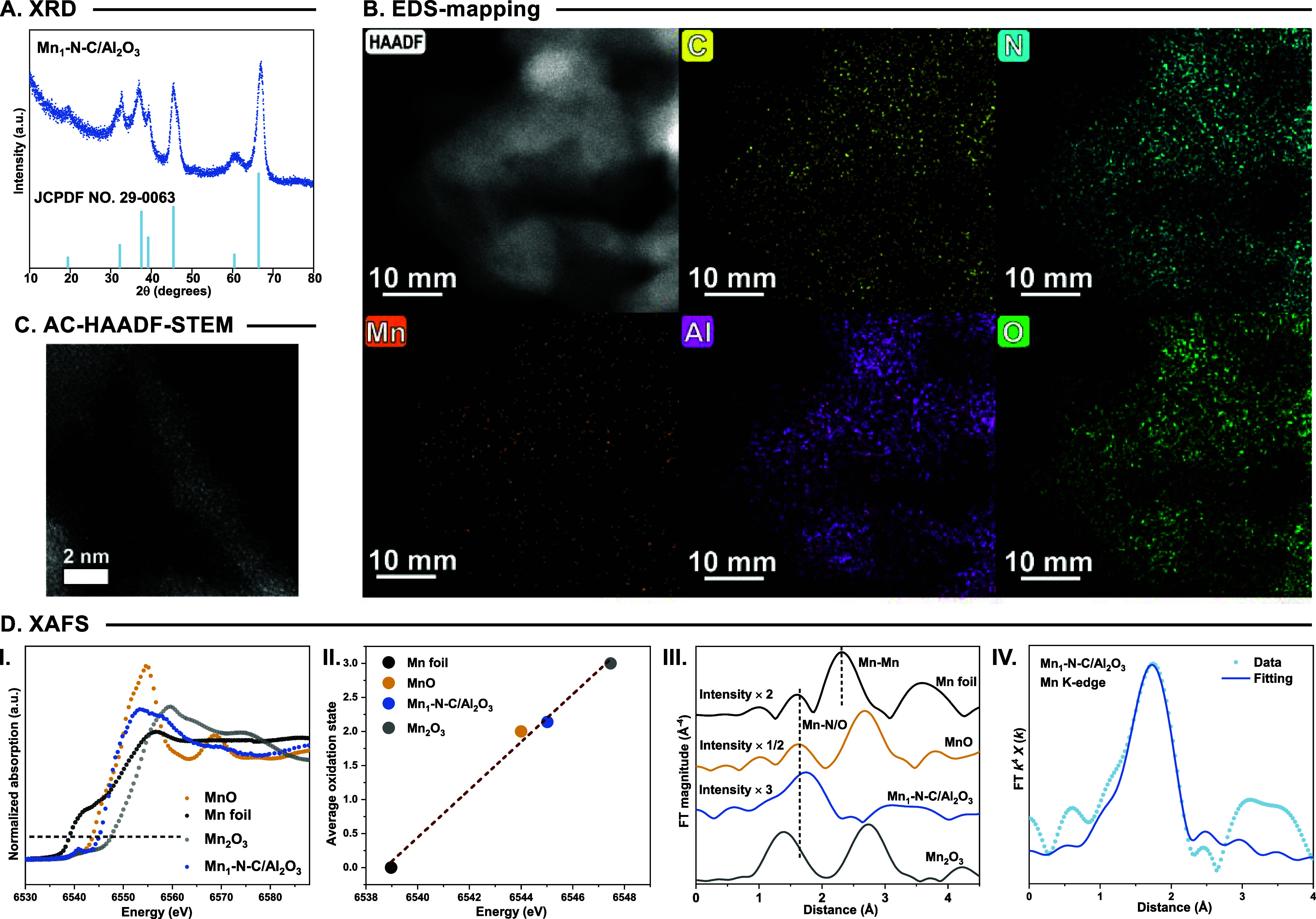
Characterizations of Mn_1_–N-C/Al_2_O_3_ catalysts: (A–C) The XRD patterns, EDS-mapping, and AC-HAADF-STEM of Mn_1_-N-C/Al_2_O_3_ catalysts. (D) XAFS analysis of Mn_1_-N-C/Al_2_O_3_. (I) The XANES spectra of Mn K-edge in Mn_1_-N-C/Al_2_O_3_ catalysts and (II) oxidation states of Mn species. (III, IV) The Fourier-transformed *k* ^2^-weighted EXAFS spectra in R-space and the corresponding fitting curves of Mn_1_-N-C/Al_2_O_3_ catalysts.

Next, X-ray photoelectron spectroscopy (XPS) and X-ray absorption fine structure (XAFS) characterizations were performed to elucidate the chemical environment of the Mn_1_ species. As displayed in Figure S5, Mn_1_-N-C/Al_2_O_3_ exhibited a doublet with 2p_3/2_ binding energy at 641.1 eV, indicating an oxidation state of Mn of about +2. From the result of Mn K-edge X-ray absorption near edge structure (XANES) spectra (Figure 2D-I), the absorption edge of Mn_1_-N-C/Al_2_O_3_ is located at the position between Mn foil and Mn_2_O_3_, like MnO. In addition, the result of the linear correlation shows that the average oxidation state of Mn_1_ single atoms is around 2.08 (Figure 2D-II), which is in good agreement with the XPS result. Notably, both Mn_1_-N-C/Al_2_O_3_ showed a distinct pre-edge peak at 6545 eV (1s to 4p_*z*_ transition), indicating the possible four-coordinated structure of the Mn_1_ single atom. On this basis, the Mn K-edge extended X-ray absorption fine structure (EXAFS) of Mn_1_-N-C/Al_2_O_3_ was investigated. As shown in Figure 2D-III, the scattering oscillation of Mn_1_–N-C/Al_2_O_3_ showed a prominent peak at 1.6 Å, which is attributed to the coordination of Mn to the light element (e.g., N, O). No distinct Mn–Mn scattering peak at about 2.2 Å was detected, indicating the single-atom dispersion of Mn, which is consistent with the AC-HAADF-STEM result. These findings were further confirmed by wavelet-transform analysis (Figure S6). Finally, least-squares EXAFS curve fitting was performed. As shown in Figure 2D-IV and Table S4, each isolated Mn atom is coordinated with four nitrogen atoms with a Mn_1_–N_4_ structure.

### Catalytic Performance and Mechanism Investigation

With Mn_1_-N-C/Al_2_O_3_ SAC in hand, the selective hydrogenation of nitroarenes, which is an important methodology to synthesize fine chemical intermediates, e.g., flavors, pharmaceuticals, and agrochemicals, was chosen as a test case. Specifically, 3-nitrostyrene (3-NS) bearing an easily reducible vinyl group next to the nitro moiety was used as a challenging model compound for the catalytic hydrogenation to the corresponding 3-aminostyrene (3-AS). Investigating the influence of key reaction parameters (temperature, pressure, solvent) on this benchmark reaction (Figure S7 and Table S5) revealed an interesting effect of traces of water on the catalytic activity of the Mn_1_-N-C/Al_2_O_3_ catalyst (see below).

At the optimized conditions (160 °C, 50 bar, 1 mL DMF, H_2_O to 3-NS ratio at ∼7.4), Mn_1_-N-C/Al_2_O_3_ exhibited good catalytic performance, allowing the complete hydrogenation of 3-NS with a selectivity of the desired product, 3-AS, above 99% (Table 1, entry 1). Even with further prolonging of the reaction, this selectivity remained stable (Figure S8), with no byproducts detected. In contrast, applying the optimized condition of our Mn_1_–N-C/Al_2_O_3_ catalyst for commercial noble-metal catalysts, such as Rh/C, Pd/C, and Pt/C, lower yields of 3-NS and poor chemoselectivity were obtained for this challenging hydrogenation process (Table 1, entries 2–4 and Table S6). Using the ratio of catalytic activity per unit time to metal price as the evaluation criteria, Mn_1_-N-C/Al_2_O_3_ is superior to the commercial catalysts mentioned. Besides, all other tested Mn­(II) salts or complexes, including MnO, manganese phthalocyanine (Mn-Pc), MnSO_4_, and Mn­(CH_3_COO)_2_, showed hardly any activity under these conditions (Table 1, entries 5–8). As for the other transition-metal-based catalysts, although the Mn_1_-N-C/Al_2_O_3_ exhibited relatively lower activity than Fe­(Co/Ni/Cu)-N-C/Al_2_O_3_ catalysts, exceptional selectivity was acquired on the Mn_1_–N-C/Al_2_O_3_ in the hydrogenation of 3-NS. Then, we choose both turnover number (TON) and turnover frequency (TOF) as the comparison indexes to make a comparison between Mn_1_-N-C/Al_2_O_3_ and its homogeneous counterparts. As shown in Table S7, the intrinsic TON on Mn_1_-N-C/Al_2_O_3_ is calculated as 60.5. Also, the TOF value is determined as 0.21 h^–1^. Both activity indexes are comparable to the homogeneous Mn complex and Fe-based heterogeneous hydrogenation catalysts (Table S8). After the TON evaluation, via treating at 400 °C in an Ar atmosphere for 1 h, the Mn_1_-N-C/Al_2_O_3_ catalyst was effectively regenerated, without leaching any Mn species into the solution (Tables S9 and 10). The decrease of the catalytic activity might be due to the adsorption of reactants or intermediates on the Mn_1_-N-C/Al_2_O_3_ surface, which can be effectively removed during the thermal treatment.

**Table 1:**
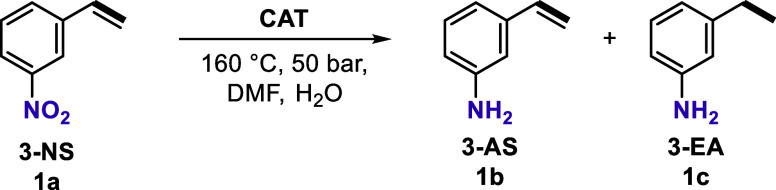
Selective Hydrogenation of 3-NS: Screening of Various Commercial Catalysts and Comparison with Mn_1_-N-C/Al_2_O_3_ ^a^

entry	catalyst	conv. (%)	**1b** (%)	**1c** (%)
1	Mn_1_-N-C/Al_2_O_3_	99	99	/
2	5%-Pt/C	30	9	21
3	5%-Rh/C	78	31	47
4	10%-Pd/C	60	7	53
5	MnO	/	/	/
6	Mn-Pc	<1	/	<1
7	MnSO_4_	/	/	/
8	Mn(CH_3_COO)_2_	/	/	/

a Reaction conditions: Entries 1, 5–8: 0.3 mmol of 3-NS, 50 mg of catalyst, 1 mL of DMF and H_2_O (H_2_O to 3-NS ratio at ∼7.4) for 18 h at 160 °C, 50 bar H_2_. Entries 2–4: 10 mg of 5%-Pt/C, 5%-Rh/C catalyst and 5 mg of 10%-Pd/C, 3 min at 160 °C, 50 bar H_2_.

After that, the role of trace amounts of water in 3-NS hydrogenation was investigated. As shown in Figure 3A and Table S11, the introduction of water remarkably improves the catalytic activity on the Mn_1_-N-C/Al_2_O_3_ catalyst. The optimal H_2_O to 3-NS ratio was 7.4, which indicated the necessity of external water addition. With a further increase of the H_2_O content, the hydrogenation activity obviously declines, suggesting the overdose of H_2_O produces side effects. When the molar ratio of H_2_O to 3-NS substrate is larger than 46.3, the catalytic activity is obviously lower than that of the water-free system. On this basis, the kinetic experiments were conducted to explore the catalytic behavior of H_2_O in the catalytic system. As shown in Figure 3B and Table S12, the reaction order of the benchmark reaction is unchanged after the introduction of H_2_O, indicating that the addition of H_2_O does not affect the activation of the catalyst material. As for the other reactant (H_2_), the reaction order increased from 0.5 to 1.0 by adding trace H_2_O into the system (H_2_O to 3-NS ratio at ∼7.4, Figure 3C and Table S13). This result demonstrates that the H_2_ activation on Mn_1_-N-C/Al_2_O_3_ SAC likely follows a heterolytic cleavage mode, such as the known homogeneous Mn­(I)-based pincer complexes.

**Figure 3:**
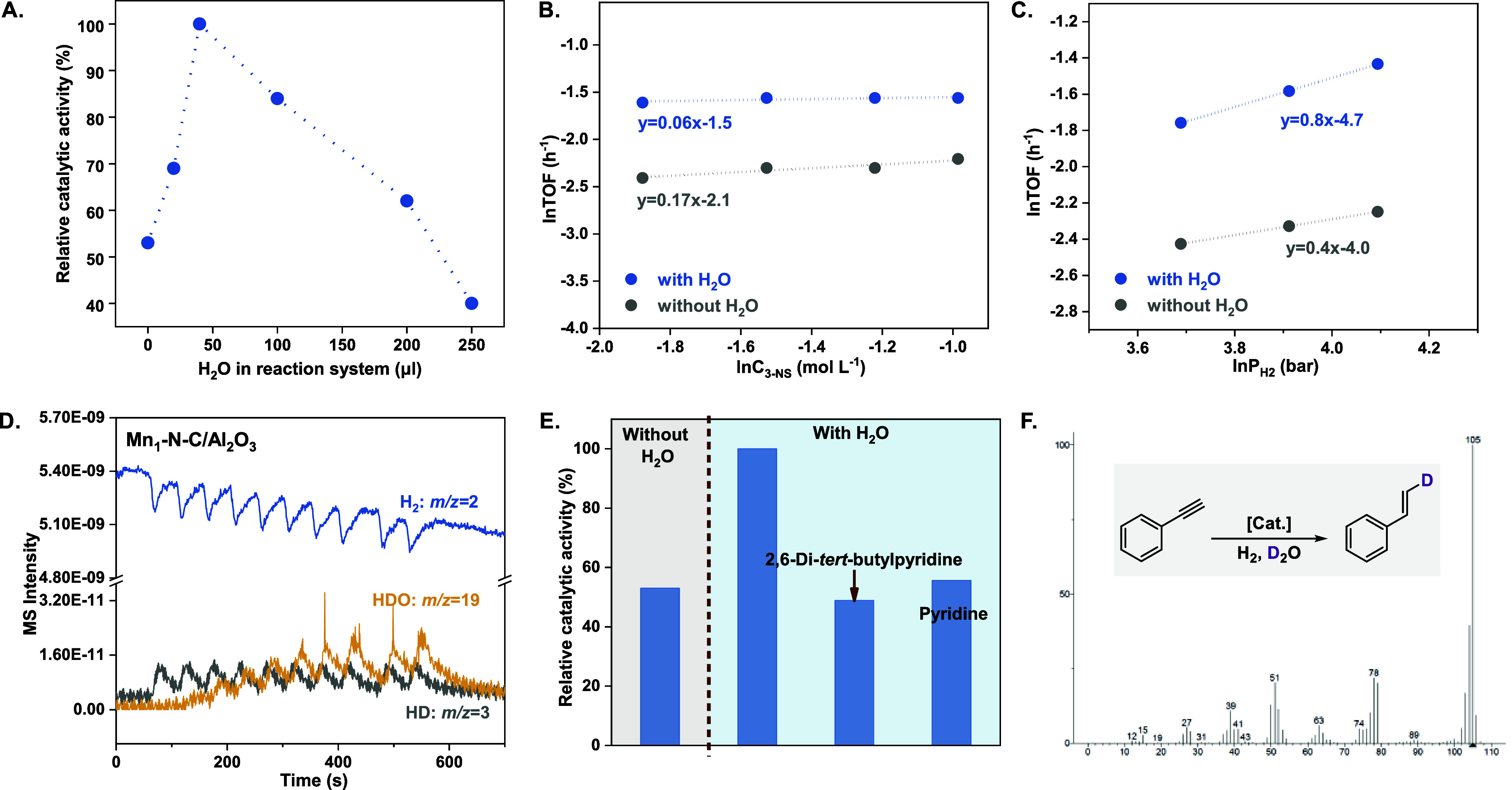
Mechanistic experiments on 3-NS hydrogenation over Mn_1_-N-C/Al_2_O_3_ SAC. (A) Relationship between H_2_O content and catalytic performance. (B) The 3-NS reaction order and (C) the H_2_ reaction order over Mn_1_-N-C/Al_2_O_3_ SAC. Reaction conditions: 50 mg of catalyst, 1 mL of DMF, and 40 μL of H_2_O (corresponding content for A) or without H_2_O, for 5 h at 160 °C, 50 bar of H_2_. (D) The H_2_-D_2_O isotopic exchange experiment on the Mn_1_-N-C/Al_2_O_3_ catalyst at 160 °C. (E) The hydrogenation activity comparison of 3-nitrostyrene on Mn_1_-N-C/Al_2_O_3_. (F) Deuterated products in the phenylacetylene hydrogenation reaction with Mn_1_-N-C/Al_2_O_3_ SAC. Reaction conditions: 50 mg of catalyst, 1 mL of DMF, and 40 μL of D_2_O for 48 h at 160 °C, 50 bar of H_2_.

Then, for an in-depth understanding of the H_2_ activation process, the H_2_-D_2_ and H_2_-D_2_O isotopic exchange experiments were conducted. As shown in Figure S10, the mass spectrometry data exhibits that the intensity of HD (*m*/*z* = 3) gradually increases from 160 to 185 °C, accompanied by the declining of H_2_ (*m*/*z* = 2) and D_2_ (*m*/*z* = 4) intensities, which confirms the activated dissociation of H_2_ on Mn_1_-N-C/Al_2_O_3_.^58^ Subsequently, the result of the H_2_-D_2_O isotopic exchange test shows that the introduction of a D_2_O pulse leads to the production of HD (*m*/*z* = 3) and HDO (*m*/*z* = 19) (Figure 3D). In contrast, the N–C/Al_2_O_3_ barely shows any capability for the H_2_ activation in H_2_-D_2_(D_2_O) isotopic exchange experiments (Figures S11 and S12), in good agreement with the catalytic performance evaluation results (Table S14). The former HD generation demonstrates that D_2_O is involved in H_2_ dissociation, and the later HDO appearance implies that the H_2_ follows a heterolytic activation mode on the Mn_1_–N_4_ single-atom site, in which the formation of HDO is derived from dissociated H^+^ and D_2_O. In the meantime, given the circumstances that the 2,6-di-*tert*-butylpyridine can selectively poison Brønsted acid, whereas pyridine displays universal toxicity for Brønsted acid and Lewis acid,^59^ we used both 2,6-di-*tert*-butylpyridine and pyridine as the toxic agents in the control experiments to investigate the role of generated H^+^ (Brønsted acid) in the hydrogenation of 3-NS (Figure 3E, see the Supporting Information for details). The blank experiment demonstrates that both 2,6-di-*tert*-butylpyridine and pyridine would not coordinate to Mn_1_ single atoms, thereby causing the decrease of activity (Table S15). With the introduction of 2,6-di-*tert*-butylpyridine, the catalytic activity apparently decreases, comparable to the no-water system. Pyridine addition would not further affect the hydrogenation activity. This result indicates that the in situ-generated H^+^ (Brønsted acid), which is produced from the heterolytic cleavage of H_2_, is the contributing factor for the improved catalytic activity. This catalytic mechanism is quite different from the H_2_O-promoted Fe­(Co/Ni/Cu)–N-C/Al_2_O_3_ hydrogenation system (Figure S13), the H_2_O-assisted adsorption strength inversion between nitro and amino groups on the Co_1_–N-C catalyst,^60^ and the way of using H_2_O to produce active hydroxyl species in CO atmosphere on α-MoC_1–*x*_-supported metal catalysts.^61^ Furthermore, it has been reported that the addition of H_2_O can promote hydrogen spillover.^62,63^ On this basis, an isotope control experiment using D_2_O was carried out. Considering that the hydrogen atoms in the resulting amino group are too reactive, they can easily be replaced by hydrogen atoms from H_2_O, phenylacetylene was chosen as the probe molecule (Figure 3F). Indeed, the use of D_2_O instead of H_2_O in the hydrogenation of phenylacetylene resulted in the formation of β-D-styrene, which clearly shows that water takes part in the hydrogenation reaction. Our proposed H_2_O-promoted hydrogenation process is also different from the recently reported H_2_O-mediated proton–electron transfer on Pt_1_/humic acid catalyst.^64^ Specifically, in the Pt_1_/humic acid system, H_2_O is indispensable for enabling a biomimetic PCET pathway, where proton transfer through a hydrogen-bond network is intrinsically coupled with electron delivery from the Pt single atom, and hydrogenation is essentially inaccessible in the absence of H_2_O.

In the meantime, the attenuated total reflectance infrared spectroscopy (ATR-IR) characterization was performed to explore the adsorption behavior of the 3-NS substrate on the Mn_1_-N-C/Al_2_O_3_ surface. As shown in Figure S14, the pure 3-NS produced two bands at 1344 and 1523 cm^–1^, which are attributed to asymmetric stretching and symmetric stretching vibrations of the nitro group, respectively.^65^ With the introduction of the Mn_1_-N-C/Al_2_O_3_ sample, one can clearly see that the 3-NS adsorbs on the catalyst surface with two nitro group bands shifting to 1350 and 1529 cm^–1^, which is similar to the adsorption behavior on γ-Al_2_O_3_. Further introducing hydrogen and increasing the temperature to 140 °C, the appearance of the new bands at 1461 cm^–1^ (–NOH) and 1494 cm^–1^ (−NH_2_) was observed on the Mn_1_-N-C/Al_2_O_3_ catalyst (Figure S15).^66^ In contrast, the same procedure on the γ-Al_2_O_3_ support did not bring about any change in the nitro adsorption bands (Figure S16). It is worth mentioning that the Al atoms in γ-Al_2_O_3_ serve as Lewis acids, and the oxygen atoms in the nitro group act as Lewis bases. The preferential adsorption of the nitro group on γ-Al_2_O_3_ can be regarded as the interaction between the Lewis acid and base, even in the presence of other reducible groups.^67^ Furthermore, we also test the 3-NS reaction order on the Mn–N–C sample, which shows a 0.8 value, much higher than the Mn_1_-N-C/Al_2_O_3_ catalyst (Figure S17 and Table S16). This result further suggests that γ-Al_2_O_3_ is reasonable for the enrichment and activation of nitro groups. Besides, the result of 3-NS adsorption content shows that both γ-Al_2_O_3_ and Mn_1_-N-C/Al_2_O_3_ samples can effectively adsorb 3-NS (Table S17), in good agreement with the ATR-IR characterization and kinetics experiment results. Taking these results together, it can be reasonably deduced that H_2_ is activated on the Mn single atoms and the nitro group is preferentially adsorbed on the γ-Al_2_O_3_ for further surface hydrogenation process in Mn_1_-N-C/Al_2_O_3_.^68^

In summary, the synergistic effect between Mn_1_-N-C and Al_2_O_3_ for the hydrogenation of nitroarenes can be clearly illustrated. First, the nitro group is preferentially adsorbed on the Al_2_O_3_ surface. Then, under anhydrous conditions, H_2_ dissociation on Mn_1_-N-C/Al_2_O_3_ likely proceeds via a homogeneous Mn–N cooperative pathway,^38^ which is similar to Co–N­(P)–C and Ni–N–C SAC systems.^69,70^ With the introduction of trace H_2_O, the hydrogen is activated on the Mn_1_ single atoms via the heterolytic activation mode. Considering that the isolated Mn­(II) atom is a Lewis acid site,^71^ and the fact that H_2_O can serve as a Lewis base site, the trace H_2_O facilitates the heterolytic cleavage of the H_2_ molecule to the H^+^ and H^–^ species,^72^ analogous to the reported H_2_O-promoted H-shuttling mechanism on Co–N–C.^73^ The generated H^+^ is interacting with H_2_O to produce H_3_O^+^, improving the catalytic hydrogenation activity through the proton transfer process on Mn_1_-N-C/Al_2_O_3_ SAC. Followed by further transfer of H^–^ species for nitro group reduction, the 3-NS was transformed into 3-AS.

### Practical Application Potential

To showcase the general applicability of Mn_1_-N-C/Al_2_O_3_ SAC, various functionalized nitroarenes were hydrogenated. As shown in Figure 4A, a series of nitroarenes, which contain alkyl, olefinyl, hydroxyl, alkoxy, carbonyl, and biphenyl groups, can be effectively converted into the corresponding substituted anilines with satisfactory yields (3b–5b, 7b, 8b, and 16b–20b). As for halide substituents, the hydrogenation of chloro-nitroarenes proceeded with high yields (11b and 12b). However, iodo-nitroarene showed poorer selectivity with this catalytic system (9b). Compared with the iodol analogue, the bromo-nitroarene shows improved catalytic performance, consistent with the weaker C–Br bond interaction (13b). Meanwhile, when the nitroarenes contain strong electron-withdrawing groups, including cyano- and para-trifluoromethyl-, good yields of the corresponding anilines were also achieved (6b and 10b). Besides, the Mn_1_-N-C/Al_2_O_3_ catalyst showed excellent selectivity for the hydrogenation of heterocyclic nitroarenes (15b). Notably, in contrast to traditional PGM-based catalysts, the Mn_1_-N-C/Al_2_O_3_ SAC displayed excellent poison-resistant capability for the selective hydrogenation of sulfur-containing nitroarenes (14b). Nevertheless, there are some limitations observed with respect to alkyne- and aldehyde-containing substrates on Mn_1_-N-C/Al_2_O_3_. In the case of terminal 3-nitrophenylacetylene, both functional groups were reduced, affording 3-aminostyrene as the sole product after 18 h. And the hydrogenation of 4-nitrobenzaldehyde resulted in a complex mixture of reduced products (Table S18).

**Figure 4:**
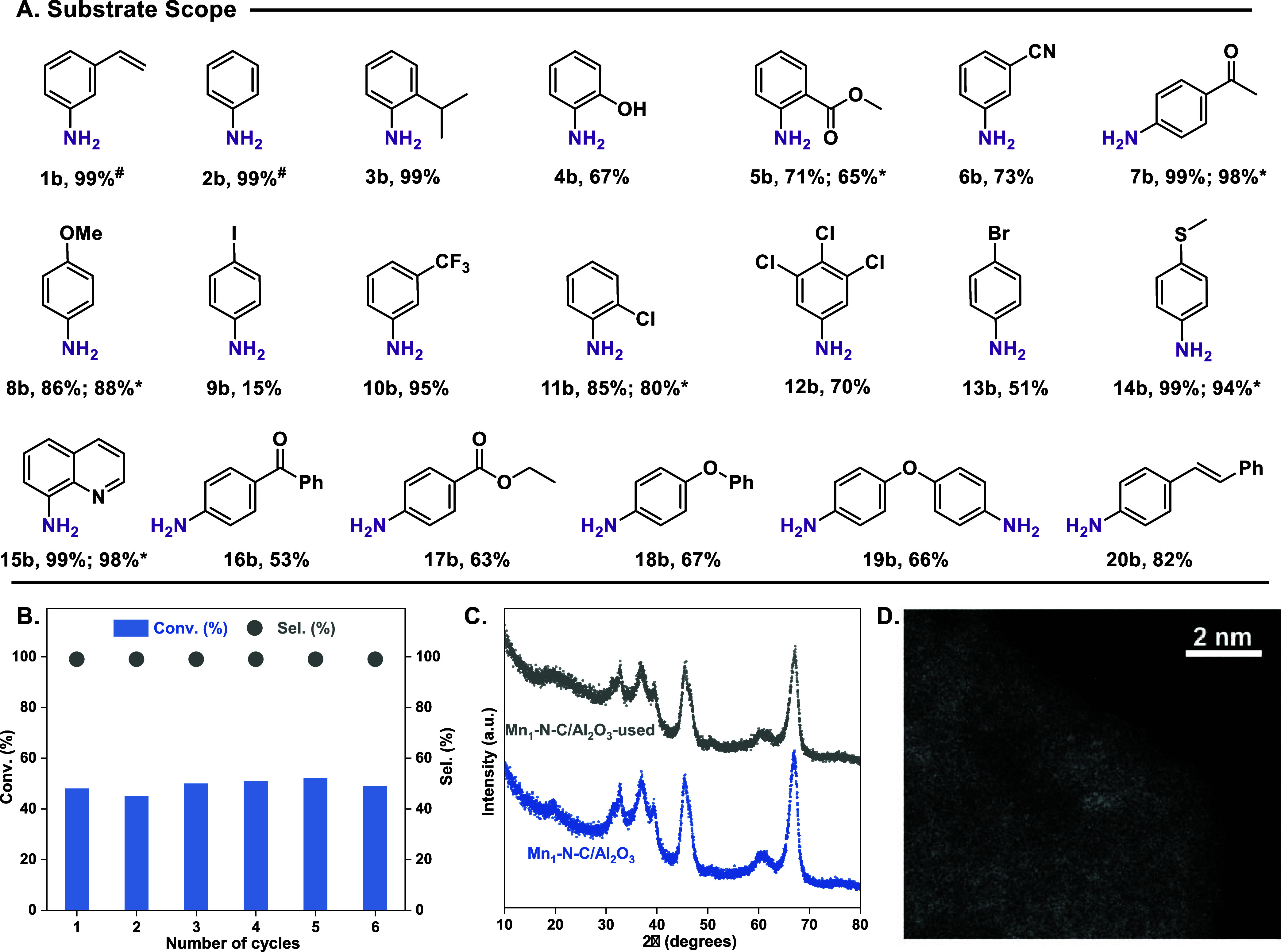
Substrate scope and reusability studies. (A) Isolated yield (^#^GC yield), 0.3 mmol of substrates, 50 mg of Mn_1_-N-C/Al_2_O_3_ catalyst, 1 mL of DMF, and 40 μL of H_2_O for 48 h at 160 °C, 50 bar of H_2_ (*reaction time for 8 h and ^#^for 18 h). (B) Recycling experiments. 0.3 mmol of 3-NS, 100 mg of catalyst, 2 mL of DMF and H_2_O (H_2_O to 3-NS ratio at ∼7.4) for 8 h at 160 °C, 50 bar of H_2_. (C) XRD pattern and (D) AC-HAADF-STEM of the used Mn_1_-N-C/Al_2_O_3_ catalysts.

While all substrates were studied under the standard reaction conditions, we also performed test reactions at a shorter reaction time (8 h) with six selected substrates. As shown in Figure 4A, similar conversions and yields were observed for most of them (5b, 7b, 8b, 11b, 14b, 15b). Finally, the stability and reusability of our optimal catalyst material were tested, which is a key feature for any application of heterogeneous catalysts. Fortunately, in the recycling experiments, Mn_1_-N-C/Al_2_O_3_ SAC showed barely any decrease in activity and selectivity over 6 cycles (Figure 4B, see also the Supporting Information for details). The Mn_1_-N-C/Al_2_O_3_ catalyst used was characterized by XRD, HAADF-STEM, and XPS. As shown in the XRD spectra (Figure 4C), the structure of the recycled material is similar to the fresh one, indicating the stability of Mn_1_-N-C/Al_2_O_3_. In the HAADF-STEM images (Figures 4D and S18), the Mn species of the used Mn_1_-N-C/Al_2_O_3_ sample can be clearly seen as isolated bright dots, showing that there is no aggregation of Mn_1_ single atoms on the support surface. The STEM and corresponding EDS analysis of the used Mn_1_-N-C/Al_2_O_3_ show a clear and uniform distribution of C, N, O, Al, and Mn species on the support (Figure S19). Regarding the oxidation state, no change in the binding energy of Mn 2p_3/2_ was observed during the reaction (Figure S20). All of these results indicate that Mn_1_-N-C/Al_2_O_3_ SAC is of high stability.

## Conclusions

In conclusion, we report here the first development of a heterogeneous Mn catalyst for selective hydrogenation reactions in organic synthesis. Using a pyrolysis preparation method, specific Mn_1_-N_4_ sites on a nitrogen–carbon-covered γ-Al_2_O_3_ surface were constructed and characterized in detail. The resulting Mn_1_-N-C/Al_2_O_3_ SAC exhibits a good catalytic performance in the hydrogenation of 20 nitroarenes. The corresponding amines were obtained with high selectivity, especially for nitrostyrene. In the hydrogenation process, the addition of H_2_O promotes the activity of the Mn_1_-N-C/Al_2_O_3_. Mechanistic control experiments suggest heterolytic activation of hydrogen. This work provides an effective approach for the design of completely new Mn-based heterogeneous hydrogenation catalysts. Here, we make it clear that we do not aim for the best TON but rather want to demonstrate that Mn can be used as a heterogeneous hydrogenation catalyst.

## Supplementary Material



## Notes

Methods and procedures of catalysts preparation, characterization, evaluation, as well as supporting figures and tables (PDF)
