# Supplementary material for: A Heterogeneous Manganese Catalyst for the Selective Hydrogenation of Nitroarenes
Source: J Am Chem Soc. 2026 Mar 26;148(13):13654–62. doi: 10.1021/jacs.5c19788 (PMC13067263; doi:10.1021/jacs.5c19788)
Supplement: Supplementary file 1 [file ja5c19788_si_001.pdf]

A Heterogeneous Manganese Catalyst for the Selective Hydrogenation of Nitroarenes

Jianglin Duan,[†] Wu Li,[†] Yujing Ren,*[,‡] Kathrin Junge,*[,†] Matthias Beller*[,†]

[†] Leibniz-Institut für Katalyse, Rostock, 18059, Germany.

[‡] Interdisciplinary Research Center of Biology & Catalysis, School of Life Science and Technology, Northwestern Polytechnical University, Xi'an, 710072, China.

Table of Contents

1. Methods

1.1 Chemicals

1.2 Preparation of catalysts

1.3 Experimental methods

1.4 Characterization methods

2. Results

2.1 Figure

2.2 Table

3. NMR

4. References

1. Methods

1.1 Chemicals

All commercial reagents were obtained from the following chemical companies: Sigma-Aldrich, Fisher Scientific, BLD pharm, TCI, and ABCR. Unless otherwise noted, the commercial reagents were used without purification. The grade of DMF solvent is summarized in Table S11.

1.2 Preparation of Catalysts.

1.2.1 Preparation of Mn_1-N-C/Al_2O_3.

$Mn(OAc)_2 \cdot 4\ H_2O$ (0.5 mmol) and 1,10-Phenanthroline (3.0 mmol) (Mn:1,10-Phenanthroline = 1:6 molar ratio) were stirred in water (30 ml) at 80 °C for 1 hour. The support γ-Al_2O_3 (492.2 mg) was then added, and the mixture was stirred at 80 °C for 15 h. Then the water was removed in vacuum and leaving behind a solid that was dried at the pump. The sample was grinded to a fine powder which was then placed in the oven at 60 °C for 10 h. The furnace was heated to 600 °C at a rate of 25 °C per minute and held for 2 hours under argon atmosphere. After the heating was switched off the oven was allowed to reach room temperature. During the whole process argon was constantly passed through the oven. The preparation procedure is as follows.

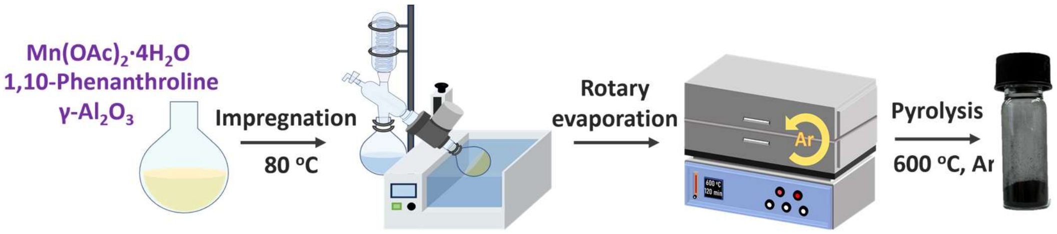

1.2.2 Preparation of M-N-C/Al_2O_3 (M= Fe, Co, Ni, Cu).

M-N-C/Al_2O_3 (M= Fe, Co, Ni, Cu) were prepared in the same method with Mn_1-N-C/Al_2O_3 sample. $Fe(OAc)_2$, $Co(OAc)_2 \cdot 4\ H_2O$, $Ni(OAc)_2 \cdot 4\ H_2O$, and $Cu(OAc)_2 \cdot\ H_2O$ as the metal precursors.

1.2.3 Preparation of M-N-C and N-C/Al_2O_3.

The preparation of M-N-C and N-C/Al_2O_3 followed the procedure of Mn_1-N-C/Al_2O_3 synthesis, except that Al_2O_3 and Mn precursors ($Mn(OAc)_2 \cdot 4\ H_2O$) were added, respectively.

1.2.4 Preparation of Mn/TiO_2, and Mn/ND.

The preparation of of Mn/TiO_2, and Mn/ND followed the similar procedure of Mn_1-N-C/Al_2O_3 synthesis. The difference is that the catalyst support was correspondingly replaced with TiO_2 and nanodiamond (ND).

1.3 Experimental methods.

1.3.1 The standard catalytic activity testing method: The catalytic activity tests were performed in a 300 ml autoclave advanced with an internal aluminum plate to include seven uniform reaction glass vials (4 ml) with cap, septum and needle. The autoclave is placed into an aluminum block as heating system to perform the reactions. 3-nitrostyrene (3-NS) and catalyst were mixed with 1 ml DMF and 40 μl H_2O in a reaction vial. After completion of the reaction time, the autoclave was cooled to room temperature. Hexadecane (30 μl) was added to the reaction mixture as internal standard and analyzed by GC.

The conversion (Conv.) of substrate and the selectivity (Sel.) of product (3-AS: 3-aminostyrene and 3-EA: 3-ethylaniline) were calculated by the following equations:

$$Conv. = \frac{n_{3-AS} + n_{3-EA}}{n_{substrate(3-NS)}}\%$$

$$Sel. = \frac{n_{3-AS}}{n_{3-AS} + n_{3-EA}}\%$$

The turnover frequency conversion (TOF) was calculated by the following equation:

$$TOF = \frac{n_{3-AS}}{n_{Mn} \times t} h^{-1}$$

1.3.2 The H_2-D_2 and H_2-D_2O isotopic exchange tests used chemisorption testing system. The H_2 and D_2 (H_2 and D_2: 20 ml/min) were introduced and accompanied programed temperature (2 °C/min from 160 °C to 185 °C). The H_2-D_2O isotopic exchange test was performed at 160 °C. The experimental procedure was continuously analyzed by GC-MS.

1.3.3 The hydrogenation activity comparison of 3-NS on Mn_1-N-C/Al_2O_3. Using the standard catalytic activity testing method for the hydrogenation activity comparison. Reaction condition: 50 mg of catalyst, 1 ml DMF and 40 μl H_2O or without H_2O, for 5 h at 160 °C, 50 bar of H_2. In this condition, the DMF solvent is stable (Figure S21).

The molar ratio of 2,6-di-*tert*-butylpyridine/pyridine to H_2O is 1:1.

1.4 Characterization methods

Scanning electron microscopy (SEM) experiments were performed with a JSM-7800F electron microscope operating at 3.0 kV. The samples were pasted on conductive carbon tape.

N_2 adsorption-desorption experiments were performed on a Micromeritics ASAP-2010 physical adsorption apparatus. Before measurement, the sample was pretreated at 200 °C for 12 h in vacuum. The specific surface area was calculated using a BrunauerEmmett-Teller (BET) method.

Inductively coupled plasma optical emission spectrometer (ICP-OES) on an Agilent 5110 instrument for the actual metal loadings.

X-ray diffraction (XRD) patterns were recorded on a PANalytical X' pert diffractometer with a Cu-Kα source. A continuous mode was used to record data in the 2θ range from 10° to 80°.

The aberration-corrected high-angle annual dark-filed scanning transmission electron microscopy (AC-HAADF-STEM) analysis was performed on a FEI Themis Z microscope equipped with spherical aberration corrector and operated at 300 kV, with a guaranteed resolution of 0.06 nm.

X-ray photoelectron spectroscopy (XPS) spectra were obtained on a Thermo ESCALAB 250 X-ray photoelectron spectrometer equipped with Al Kα excitation source and with C as internal standard (C 1s = 285.0 eV).

The X-ray absorption fine structure (XAFS) spectra, including X-ray absorption near edge structure (XANES) and extended X- ray absorption fine structure (EXAFS) at Mn K-edge of the samples were recorded at the beamline 14W of Shanghai Synchrotron Radiation Facility (SSRF) in China.

The attenuated total reflection infrared (ATR-IR) spectroscopy was acquired with a Thermo Scientific Nicolet iS50 spectrometer equipped with a mercury cadmium telluride (MCT) detector at a resolution of 4 cm^{-1}. The samples were dispersed in ethanol. Then, the suspension was dropwise introduced onto the diamond crystal surface on the instrument at the 65 ^{o}C and dried at 100 ^{o}C. Before data collection, background spectrum was recorded.

Nuclear Magnetic Resonance (NMR) spectroscopy was recorded on Bruker Avance 300 (300 MHz) or 400 (400 MHz) NMR spectrometer. The chemical shifts (δ) are reported in parts per million (ppm) and coupling constants (J) in hertz (Hz).

2. Results

2.1 Figure

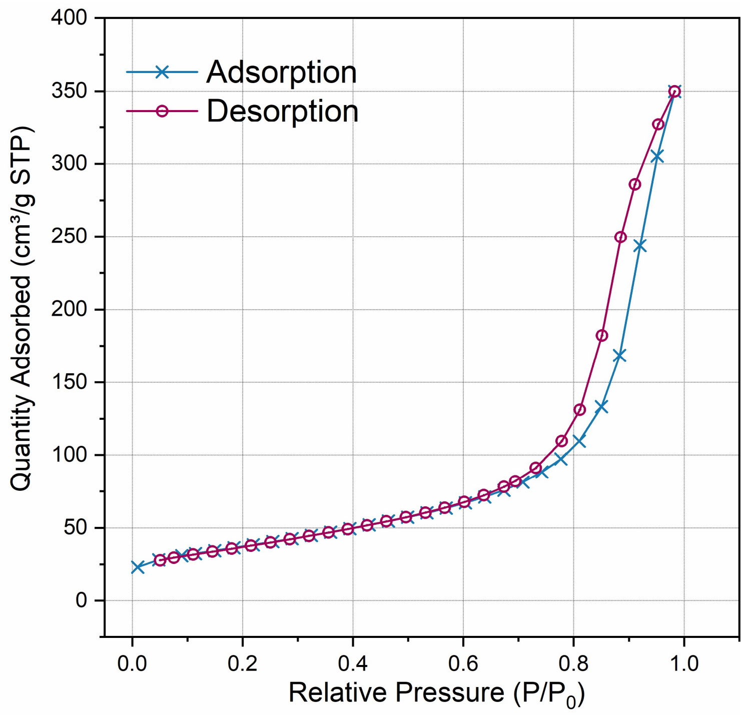

Fig. S1. Nitrogen adsorption and desorption isotherms of Mn_1-N-C/Al_2O_3.

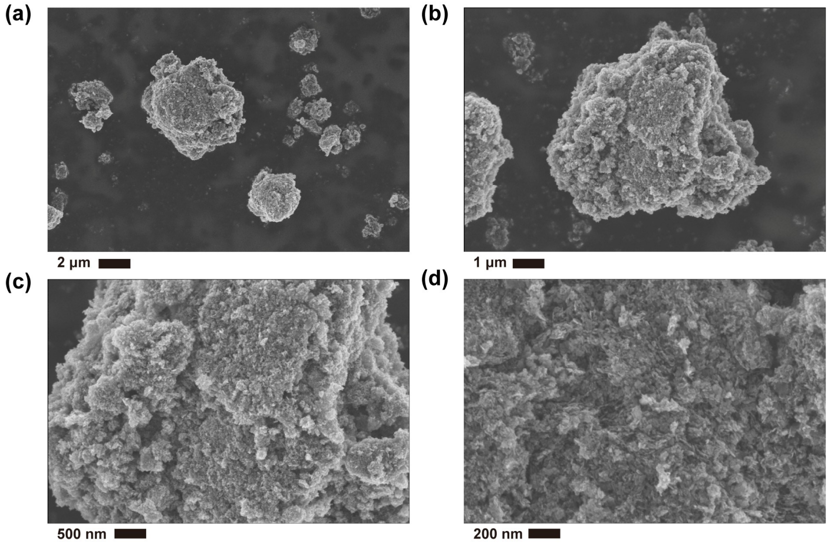

Fig. S2 SEM images of Mn_1-N-C/Al_2O_3.

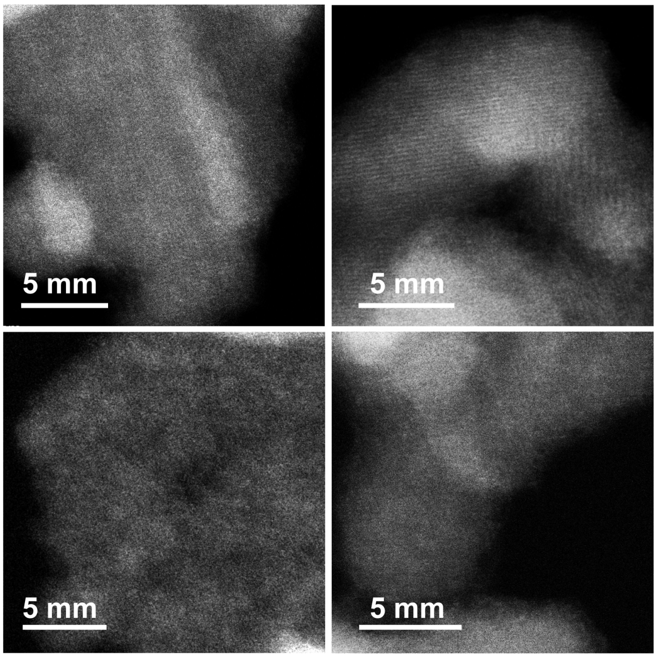

Fig. S3 STEM images of Mn_1-N-C/Al_2O_3.

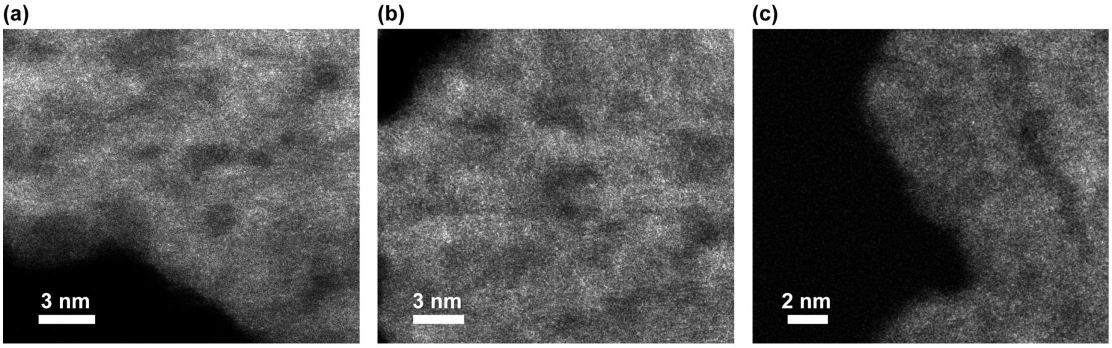

Fig. S4 AC-HAADF-STEM images of Mn_1-N-C/Al_2O_3.

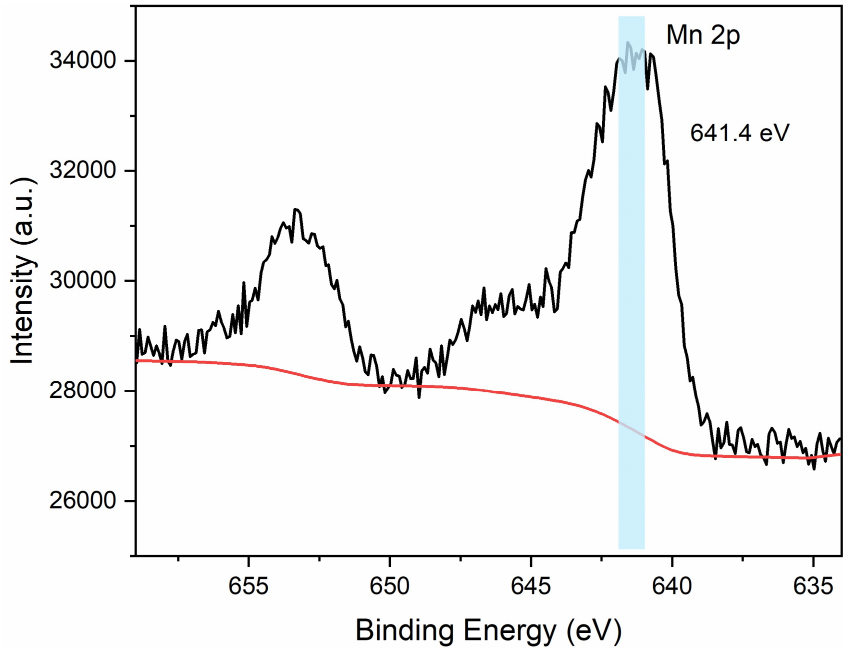

Fig. S5 XPS of Mn_1-N-C/Al_2O_3.

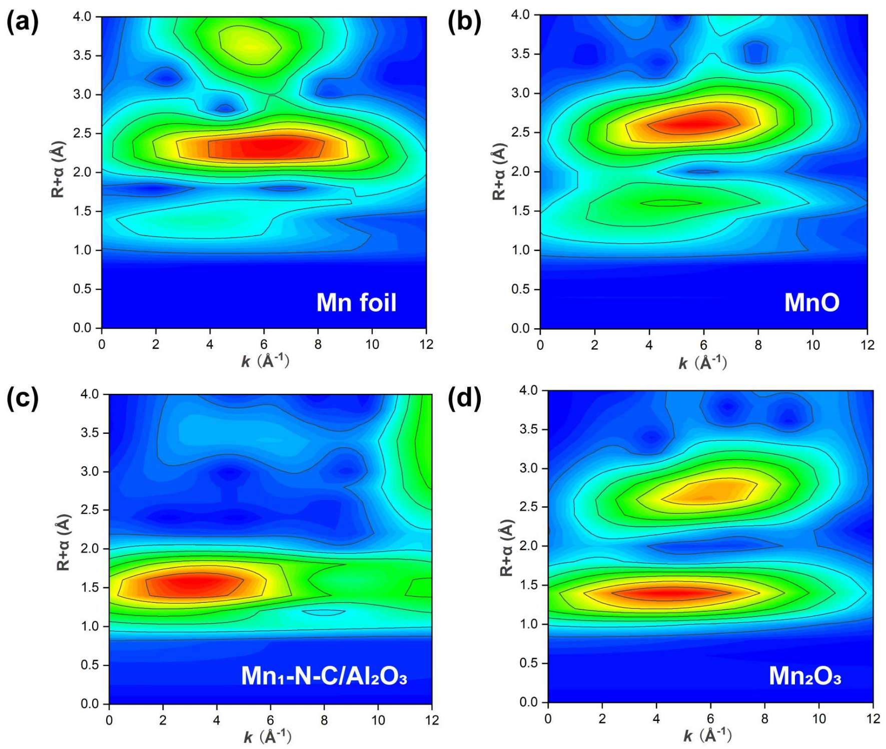

Fig. S6 Wavelet-transform analysis of (a) Mn foil, (b) MnO, (c) Mn_1-N-C/Al_2O_3 and (d) Mn_2O_3.

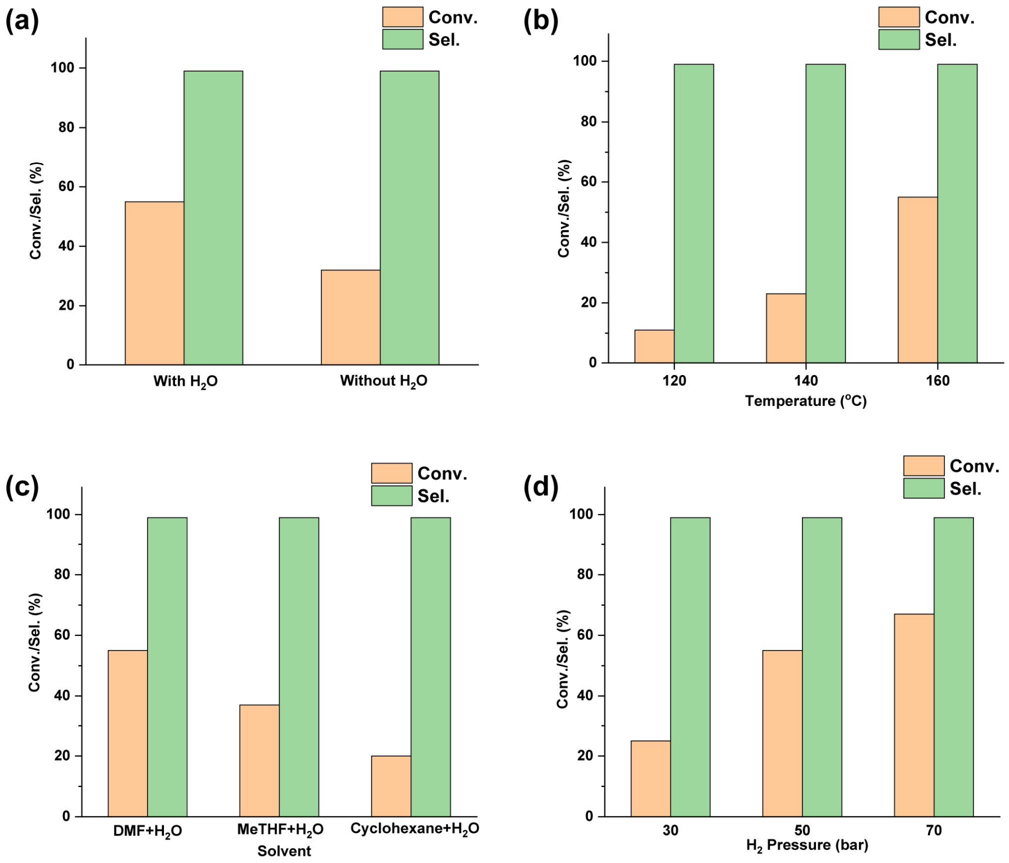

Fig. S7 Screening reaction conditions for hydrogenation of 3-NS over Mn_1-N-C/Al_2O_3 catalyst. Reaction time: 10 h. (a) 160 ^oC, H_2 50 bar, 1 ml DMF with/without H_2O, (b) H_2 50 bar, 40 μl H_2O + 1 ml DMF, (c) 160 ^oC, H_2 50 bar, 40 μl H_20 + 1 ml organic solvent and (d) 160 ^oC, 40 μl H_2O + 1 ml DMF.

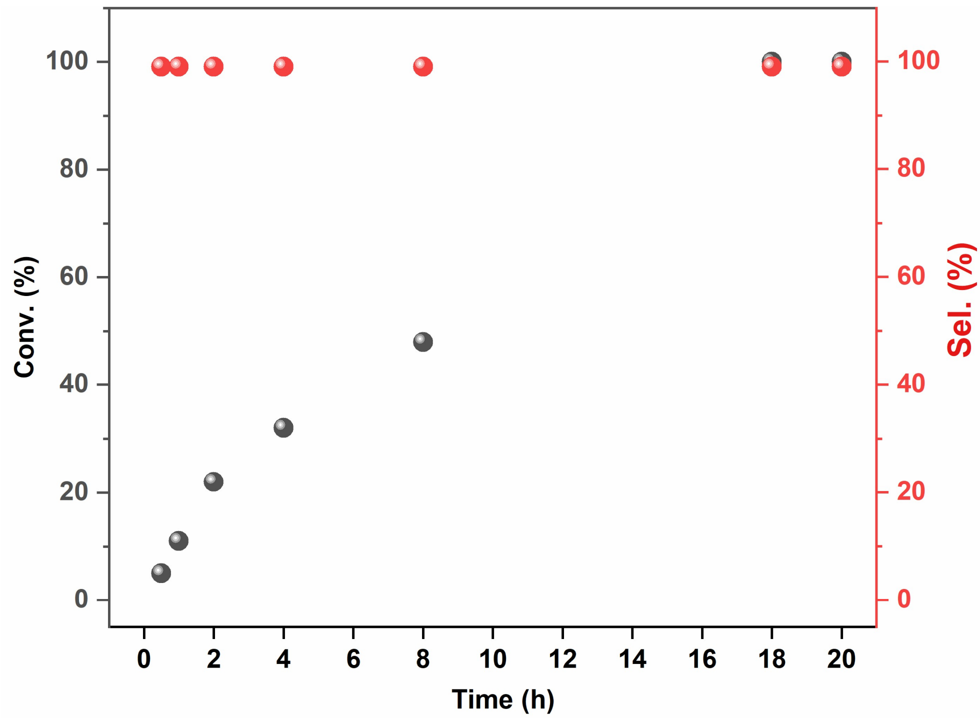

Fig. S8 Evolution of conversion and selectivity with reaction time in hydrogenation of 3-NS over Mn_1-N-C/Al_2O_3.

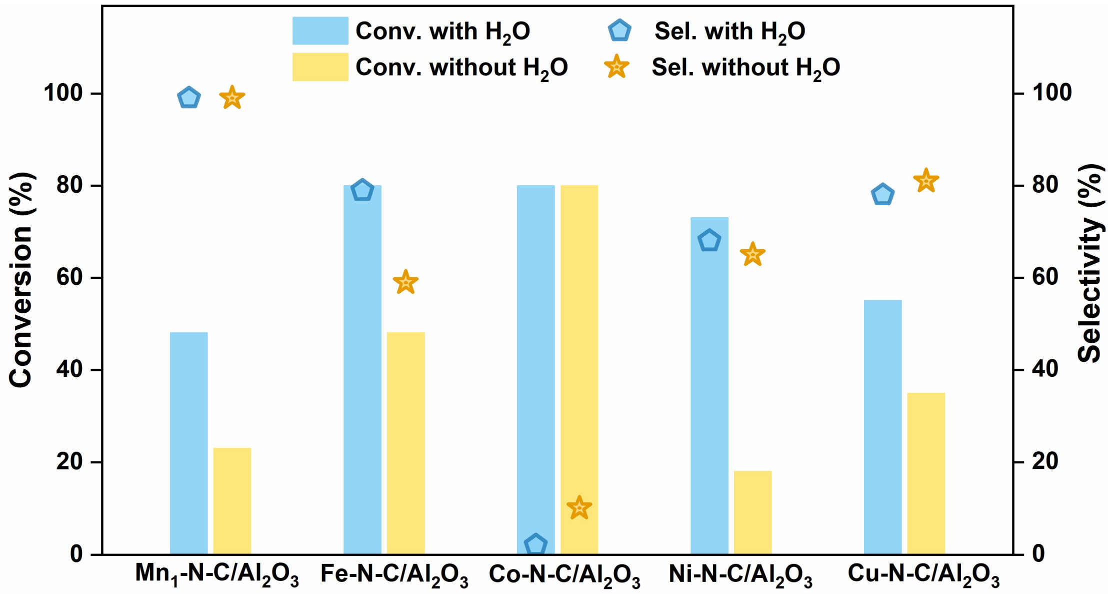

Fig. S9. Comparison of the catalytic performance of 3-NS hydrogenation. Reaction conditions: 0.3 mmol 3-NS, 50 mg sample, 1 ml DMF and with or without H_2O (H_2O to 3-NS ratio at ~7.4), 160 °C, 50 bar H_2. Reaction time: Mn_1-N-C/Al_2O_3 for 8h, Fe-N-C/Al_2O_3 for 4h, Co-N-C/Al_2O_3 for 2h, Ni-N-C/Al_2O_3 and Cu-N-C/Al_2O_3 for 5h.

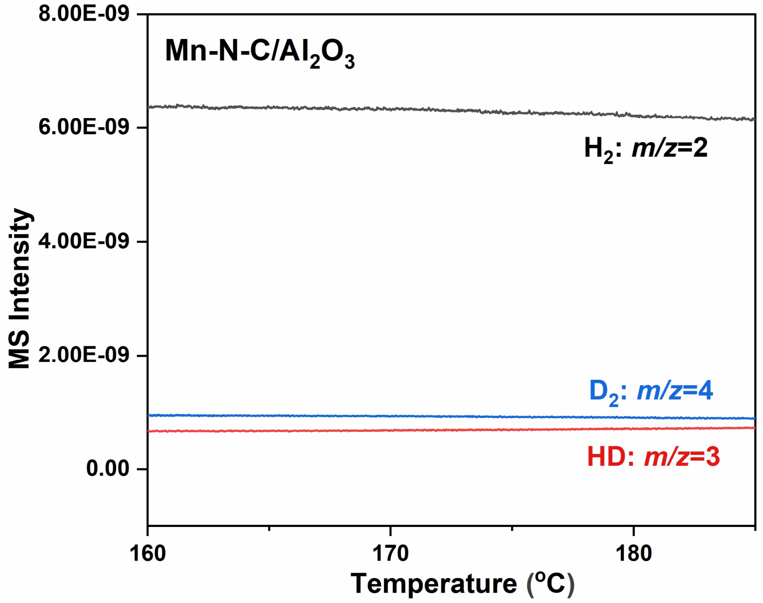

Fig. S10. The H_2-D_2 isotopic exchange experiment on Mn_1-N-C/Al_2O_3 catalyst from 160 °C to 185 °C.

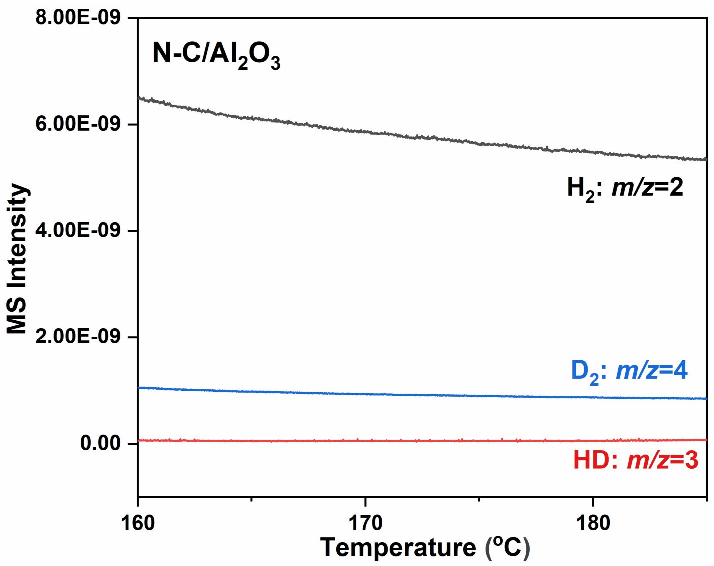

Fig. S11. The H_2-D_2 isotopic exchange experiment on N-C/Al_2O_3 sample from 160 ^oC to 185 ^oC.

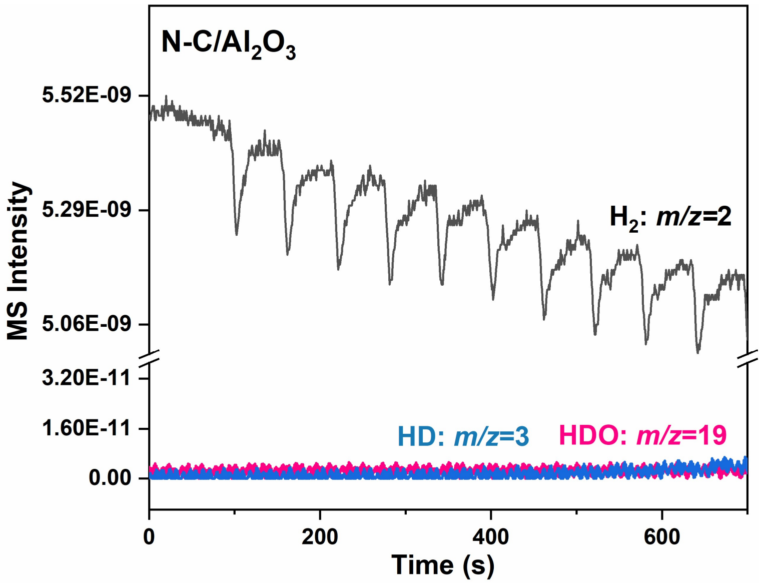

Fig. S12. The H_2-D_2O isotopic exchange experiment on N-C/Al_2O_3 sample at 160 ºC.

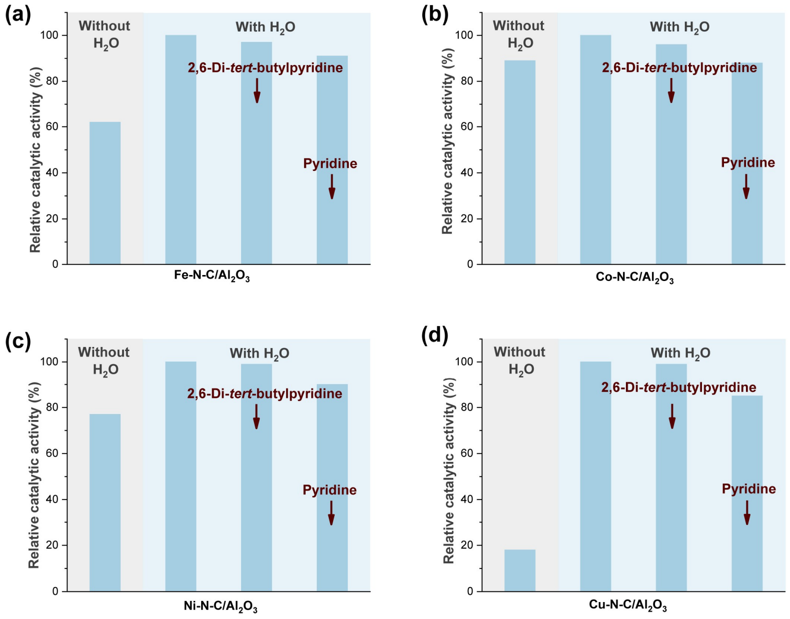

Fig. S13. The poisoning experiments for the hydrogenation of 3-NS on Fe(Co/Ni/Cu)-N-C/Al_2O_3.

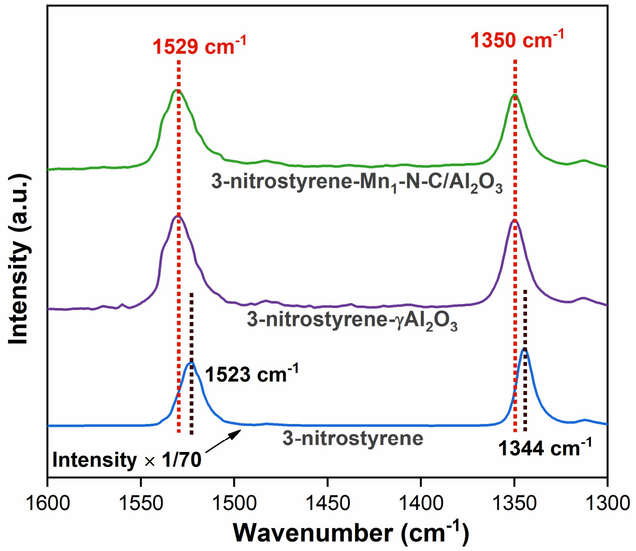

Fig. S14. ATR-IR spectra of 3-nitrostyrene (3-NS) on γ-Al_2O_3 and Mn_1-N-C/Al_2O_3 catalyst.

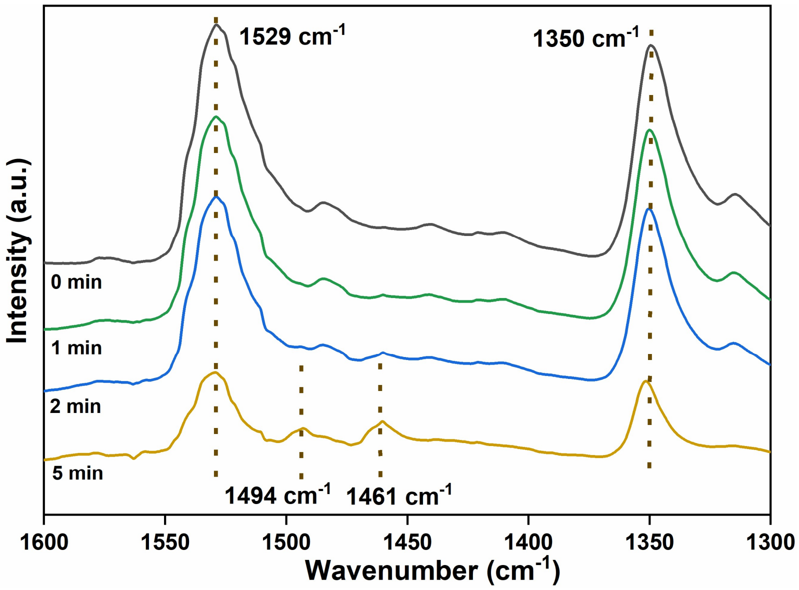

Fig. S15. The in-situ ATR-IR spectra of Mn_1-N-C/ Al_2O_3 catalyst for the hydrogenation of 3-NS.

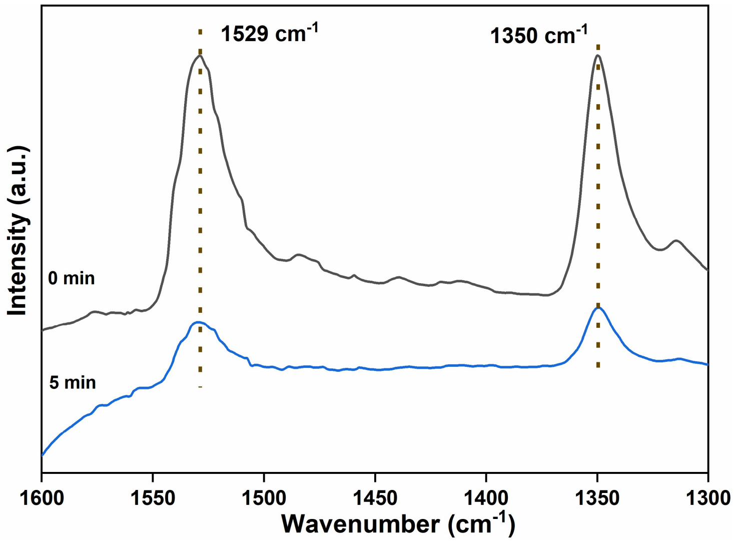

Fig. S16. The in-situ ATR-IR spectra of N-C/ Al_2O_3 sample for the hydrogenation of 3-NS.

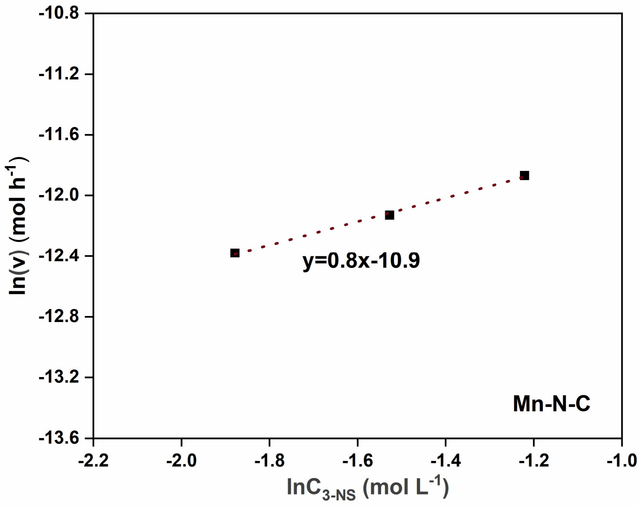

Fig. S17. The 3-NS reaction order for the hydrogenation of 3-NS on Mn-N-C sample.

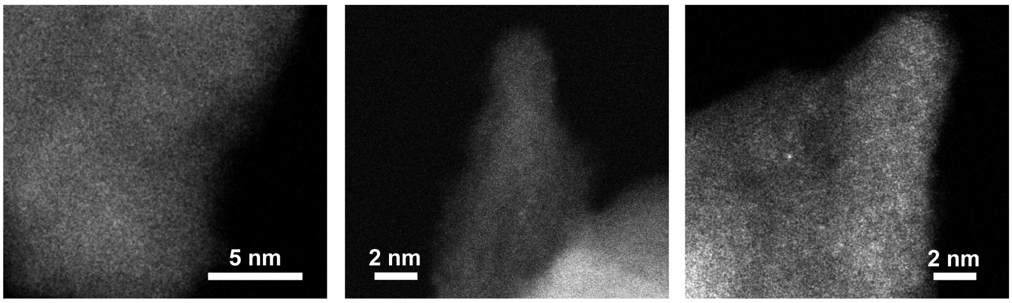

Fig. S18. The HAADF-STEM images of used Mn_1-N-C/Al_2O_3.

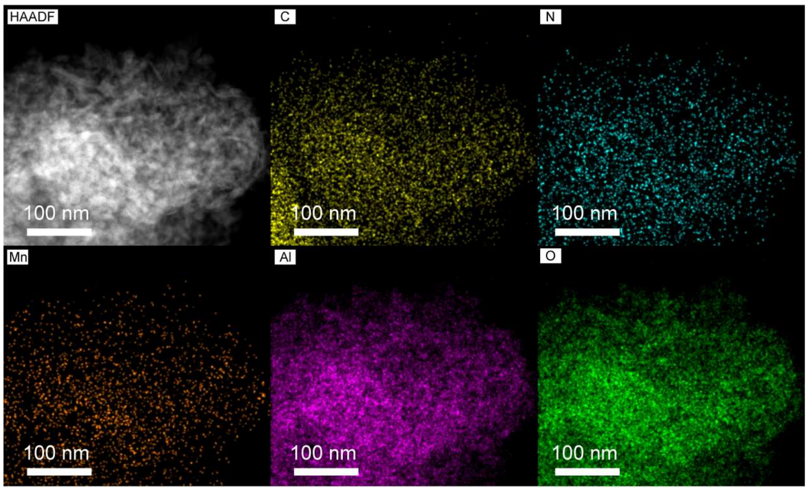

Fig. S19. The STEM and corresponding EDS analysis of the used Mn_1-N-C/Al_2O_3.

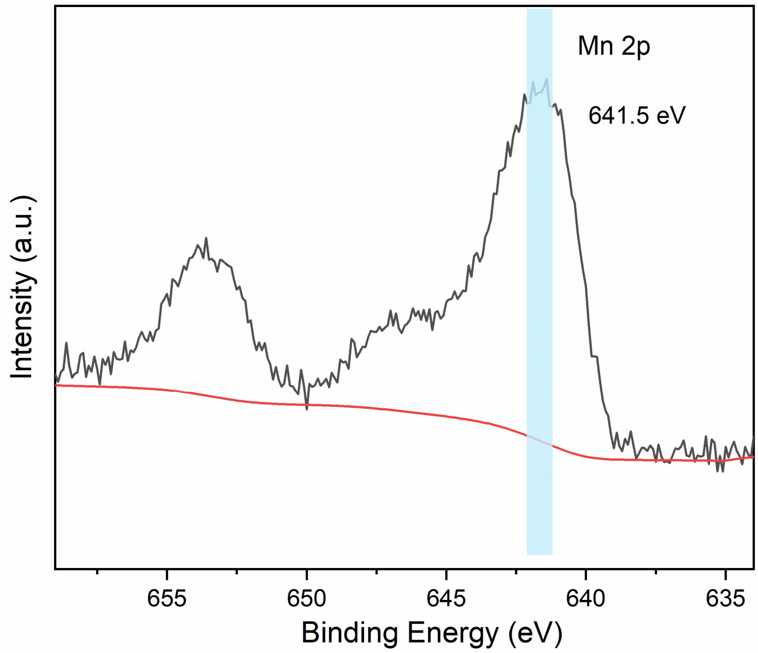

Fig. S20. XPS of used Mn_1-N-C/Al_2O_3.

Before

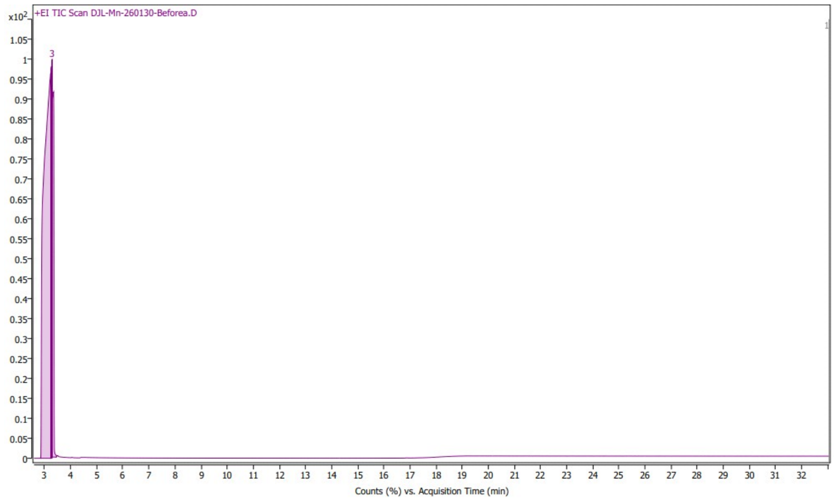

After

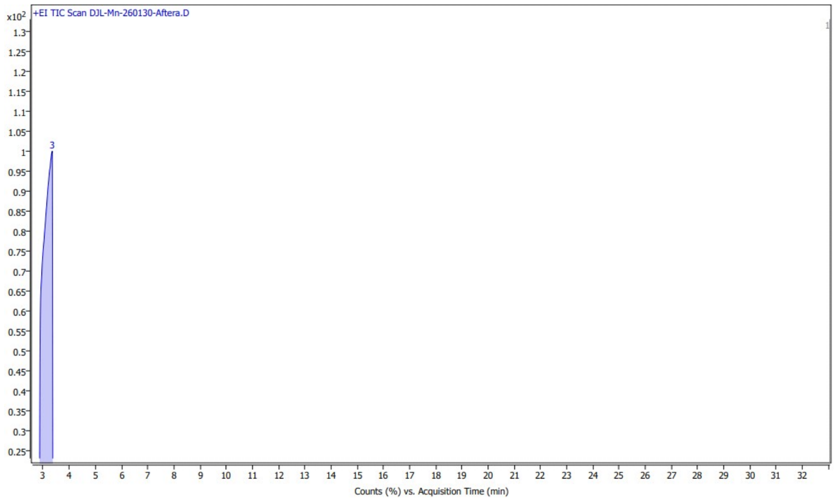

Figure S21. GC-MS spectra of DMF before and after treatment under the reaction conditions.

2.2 Table

Table S1. Parameters for catalyst preparation.

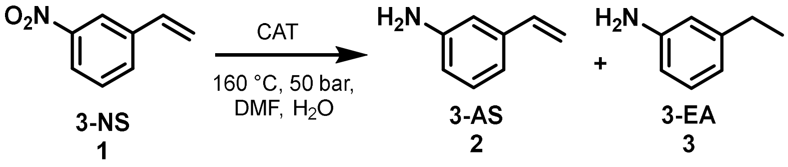

Entry	Sample	Pyrolysis Temperature	3-AS (%)	3-EA (%)	
1	Mn/Al_2O_3-400	400 °C	/	/	
2	Mn/Al_2O_3-600	600 °C	99	/	√
3	Mn/Al_2O_3-800	800 °C	37	62	
Entry	Sample	Mn salts	3-AS (%)	3-EA (%)	
4	$Mn(OAc)_2/Al_2O_3$	$Mn(OAc)_2\cdot 4\ H_2O$	99	/	√
5	$MnCl_2/Al_2O_3$	$MnCl_2\cdot 4\ H_2O$	22	46	
6	$Mn(NO_3)_2/Al_2O_3$	$Mn(NO_3)_2\cdot 4\ H_2O$	75	12	
Entry	Sample	Ligands	3-AS (%)	3-EA (%)	
7	Mn-1/Al_2O_3	melamine	89	5	
8	Mn-2/Al_2O_3	1,10-phenanthroline	99	/	√
9	Mn-3/Al_2O_3	phenylalanine	/	/	
Entry	Sample	Support			
10	Mn/Al_2O_3	γ-Al_2O_3	99	/	√
11	Mn/TiO_2	TiO_2	13	35	
12	Mn/ND	ND	68	/	

Reaction conditions: 0.3 mmol 3-NS, 100 mg catalyst, 2 ml DMF and trance H_2O (H_2O to 3-NS ratio at ~7.4), 18 hours, 160 °C, 50 bar of H_2

Table S2. BET surface area of Mn_1-N-C/Al_2O_3 SAC.

Sample	S_{BET} (m^2/g)
Mn_1-N-C/Al_2O_3	135.3

Table S3. ICP results of Mn_1-N-C/Al_2O_3 SAC.

Metal	Mass Fraction
Mn	2.86%
Fe	0.00%
Co	0.00%
Ni	0.01%
Pd	0.00%
Pr	0.00%
Pt	0.00%
Re	0.00%
Rh	0.00%
Ru	0.00%

Table S4. The best-fitted EXAFS result of Mn_1-N-C/Al_2O_3 SAC.[a]

Sample	Shell	CN	R (Å)	σ^2 (10^{-2} Å^2)	ΔE_0 (eV)	r-factor (%)
Mn_1-N-C/Al_2O_3	Mn-N	3.6	2.15	1.3	3.1	0.15

[a] CN is the coordination number for the absorber-backscatterer pair, R is the average absorber-backscatterer distance, σ^2 is the Debye-Waller factor, and ΔE_0 the inner potential correction. The data range used for data fitting in k-space (Δk) and R-space (ΔR) are 3.2-10.0 Å^{-1} and 1.0-2.2 Å, respectively. For Mn K-edge EXAFS spectra fitting the S_0^2 value is 0.70.

Table S5. The catalytic performance of 3-NS hydrogenation on γ-Al_2O_3.

Sample	Conversion (%)
γ-Al_2O_3	/

Reaction conditions: 0.3 mmol 3-NS, 50 mg γ- Al_2O_3, 1 ml DMF and trace H_2O (H_2O to 3-NS ratio at ~7.4), 18 h, 160 °C, 50 bar of H_2.

Table S6. The catalytic performance of commercial catalysts.

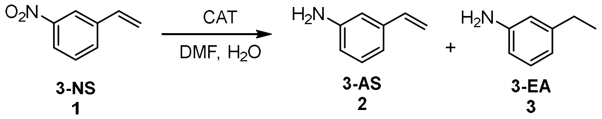

Entry	Catalyst	T (°C)	P (bar)	Conv. (%)	2 (%)	3 (%)
1	5%-Pt/C	50	5	14	/	14
2	5%-Rh/C	50	5	78	5	73
3	10%-Pd/C	50	5	39	/	39

Reaction conditions: 0.3 mmol of 3-NS, 50 mg of catalyst, 1 ml of DMF and H_2O (H_2O to 3-NS ratio at ~7.4). 10 mg of 5%-Pt/C, 5%-Rh/C catalyst and 5 mg 10%-Pd/C.

Table S7. The catalytic activity evaluation for the hydrogenation of 3-NS on Mn_1-N-C/Al_2O_3.

Catalyst	TOF[a]	TON[b]
Mn_1-N-C/Al_2O_3	0.21 h^{-1}	60.5

[a]Reaction condition: 0.3 mmol 3-NS, 50 mg catalyst, 2 ml DMF and trace H_2O (H_2O to 3-NS ratio at ~7.4), 50 bar H_2, 160 °C.

[b]Reaction condition: 2 mmol 3-NS, 50 mg catalyst, 2 ml DMF and trace H_2O (H_2O to 3-NS ratio at ~7.4), 50 bar H_2, 160 °C.

For determining the intrinsic TON of Mn_1-N-C/Al_2O_3, we increased the 3-NS concentration and prolonged the reaction time until the catalyst lost hydrogenation activity.

Table S8. The comparison of catalytic hydrogenation activity between Mn_1-N-C/Al_2O_3 and non-noble-based catalysts.

Entry	Catalyst	Substrate	Reaction Condition	Additive	TON	TOF (h^{-1})	Ref
1	Mn Cat	Ester	30 bar H_2, 80 °C	KO^{t}Bu	140	/	1
2	Mn Cat	Nitrobenzene	50 bar H_2, 130 °C	KO^{t}Bu	11.8	0.49	2
3	Mn Cat	4-Fluoroacetophenone	50 bar H_2, 25 °C	/	/	1.32	3
4	Mn Cat	(+)-Sclareolide	50 bar H_2, 90 °C	KO^{t}Bu	1776	/	4
5	Mn Cat	Athylene carbonate	30 bar H_2, 120 °C	NaO^{t}Bu	240	/	5
6	Ni–N–C-700$^{\#}$	4-Chloronitrobenzene	30 bar H_2, 120 °C	/	/	8.4	6
7	Ni800NH_3-$H_2$$^{\#}$	3-Nitrostyrene	30 bar H_2, 80 °C	/	/	37.6	7
8	Ni/C_{60}-Ac-B-4*	Nitrobenzene	20 bar H_2, 110 °C	/	/	6.3	8
9	Ni-P*	3-Nitrostyrene	10 bar H_2, 70 °C	/	/	88	9
10	Co800$NH_3$$^{\#}$	3-Nitrostyrene	30 bar H_2, 80 °C			152.3	7
11	Co@Co–N–C@SBA-15*	4-nitrostyrene	5 bar H_2, 110 °C	/	50	/	10
12	Co-N-C$^{\#}$	3-Nitrostyrene	10 bar H_2, 80 °C	/	/	2.8	11
13	Co@mesoNC$^{\#}$	Nitrobenzene	30 bar H_2, 110 °C	/	/	42	12
14	CoOx@NCNTs*	Nitrobenzene	30 bar H_2, 110 °C	/	/	8.3	13
15	Fe/N-C-500*	Nitrobenzene	40 bar H_2, 120 °C	/	8.3	0.6	14
16	Fe800$NH_3$$^{\#}$	3-Nitrostyrene	30 bar H_2, 80 °C	/	/	3.5	7
17	Mn_1-N-C/ $Al_2O_3$$^{\#}$	3-Nitrostyrene	50 bar H_2, 160 °C	/	60.5	0.21	This work

Entries 1-5: Homogenous catalysts

Entries 6-13: Heterogeneous catalysts

*: Nano catalysts

$^{\#}$: Single-atom catalyst

Table S9. The catalytic activity of Mn_1-N-C/Al_2O_3 for the hydrogenation of 3-NS after the regeneration.[a]

Catalyst	TOF[b]
Mn_1-N-C/Al_2O_3	0.21 h^{-1}
Mn_1-N-C/Al_2O_3-regenerated	0.19 h^{-1}

[a]Regeneration condition: 400 °C, Ar, 1 h.

[b]Reaction condition: 0.3 mmol 3-NS, 50 mg catalyst, 2 ml DMF and trace H_2O (H_2O to 3-NS ratio at ~7.4), 50 bar H_2, 160 °C.

Table S10. The ICP result of Mn_1-N-C/Al_2O_3 and Mn_1-N-C/Al_2O_3-used catalyst.

Sample	Mn loading wt%
Mn_1-N-C/Al_2O_3	2.86%
Mn_1-N-C/Al_2O_3-used	2.85%

Table S11. The catalytic activity for the hydrogenation of 3-NS on Mn_1-N-C/Al_2O_3.

Solvent	DMF grade	TOF
DMF	TCI, analytical grade	0.10 h^{-1}
Anhydrous DMF	TCI, analytical grade, 3 Å molecular sieves argon atmosphere	0.10 h^{-1}
$MgSO_4$-treated anhydrous DMF	TCI, analytical grade, 3 Å molecular sieves argon atmosphere	0.09 h^{-1}

Reaction condition: 0.3 mmol 3-NS, 50 mg Mn_1-N-C/Al_2O_3 catalyst, 1 ml DMF, 50 bar H_2, 160 °C.

Table S12. The catalytic activity for the hydrogenation of 3-NS with different 3-NS concentration on Mn_1-N-C/Al_2O_3 SAC.

$C_{3\text{-NS}}$ (mol/L)	TOF (h^{-1})	
	With H_2O	Without H_2O
0.15	0.20	0.09
0.22	0.21	0.10
0.30	0.21	0.10
0.38	0.21	0.11

Reaction conditions: 50 mg catalyst, 1 ml DMF and trace H_2O (H_2O to 3-NS ratio at ~7.4), 5 h, 160 °C, 50 bar of H_2.

Table S13. The catalytic activity for the hydrogenation of 3-NS with different H_2 pressure on Mn_1-N-C/Al_2O_3 SAC.

H_2 pressure	TOF (h^{-1})	
(bar)	With H_2O	Without H_2O
40	0.17	0.09
50	0.21	0.10
60	0.24	0.11

Reaction conditions: 0.3 mmol 3-NS, 50 mg catalyst, 1 ml DMF and trace H_2O (H_2O to 3-NS ratio at ~7.4), 5 h, 160 °C.

Table S14. The catalytic activity of 3-NS hydrogenation on N-C/Al_2O_3 sample.[a]

Sample	Conv. (5h)	Conv. (18h)
N-C/Al_2O_3	/	< 1%

[a]Reaction condition: 0.3 mmol 3-NS, 50 mg sample, 1 ml DMF and trace H_2O (H_2O to 3-NS ratio at ~7.4), 160 °C, 50 bar H_2.

Table S15. The catalytic activity for the hydrogenation of 3-NS.

Poisoning agent	TOF
Without toxic agent	0.10 h^{-1}
pyridine	0.09 h^{-1}
2,6-di-tertbutylpyridine	0.09 h^{-1}

Reaction condition: 0.3 mmol 3-NS, 50 mg Mn_1-N-C/Al_2O_3 catalyst, 1 ml DMF, 50 bar H_2, 160 °C.

Table S16. The catalytic hydrogenation activity of Mn-N-C with different 3-NS concentration.[a]

$C_{3\text{-NS}}$ (mol/L)	v (mol/h)[b]
0.22	4.2×10^{-7}
0.30	5.4×10^{-7}
0.38	7.0×10^{-7}

[a]Reaction condition: 10 mg sample, 1 ml DMF, trace H_2O (H_2O to 3-NS ratio at ~7.4), 5 h, 160 °C, 50 bar H_2.

[b]The catalytic activity (v) is determined according to the following equation: $v = \frac{n_{product}}{t}$ (mol/h)

Table S17. The adsorption capacity of γ-Al_2O_3 and Mn_1-N-C/Al_2O_3 for 3-NS substrate.[a]

Sample	Adsorption ratio (3-NS (mmol)/sample (g))
γ-Al_2O_3	1.7 mmol/g
Mn_1-N-C/ Al_2O_3	1.9 mmol/g

[a]Adsorption condition: 0.3 mmol 3-NS, 50 mg sample, stirring for 18 h, 160 °C, 50 bar N_2.

Table S18. Hydrogenation of 3-nitrophenylacetylene and 4-nitrobenzaldehyde on Mn_1-N-C/Al_2O_3.

Substrate	Product	Conv. (%)	Sel. (%)
3-nitrophenylacetylene [a]	3-aminostyrene	> 99	> 99
4-nitrobenzaldehyde	4-aminobenzaldehyde	6	8.3
	4-nitrobenzyl alcohol	48	-
	4-aminobenzyl alcohol	15	-
	4-toluidine	3	-
	Total	72	-

Reaction condition: 50 mg of Mn_1-N-C/Al_2O_3 catalyst, 1 ml DMF and 40 µl H_2O at 160 °C, 50 bar of H_2. Conversion and selectivity were determined by GC analysis using GC–MS for product identification.

3 NMR

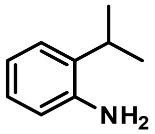

(3b)

1H NMR (300 MHz, $CDCl_3$) δ 7.11 (dd, J = 7.6, 1.0 Hz, 1H), 6.96-6.70 (m, 1H), 6.84 – 6.77 (m, 1H), 6.70 (dd, J = 7.9, 1.3 Hz, 1H), 3.66 (s, br, 2H), 2.98 – 2.86 (m, 1H), 1.29 (d, J = 6.8 Hz, 6H). 13C NMR (75 MHz, $CDCl_3$) δ 143.42, 132.76, 126.64, 125.51, 119.11, 115.92, 27.75, 22.39.

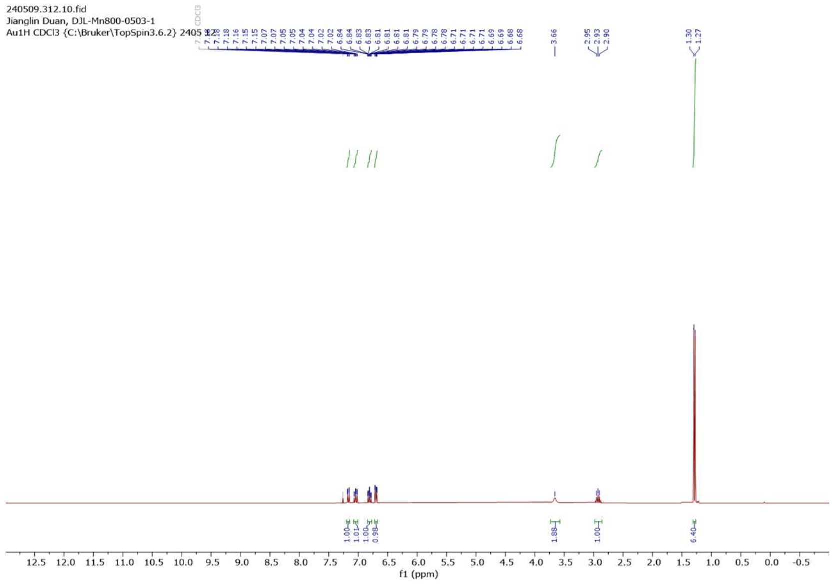

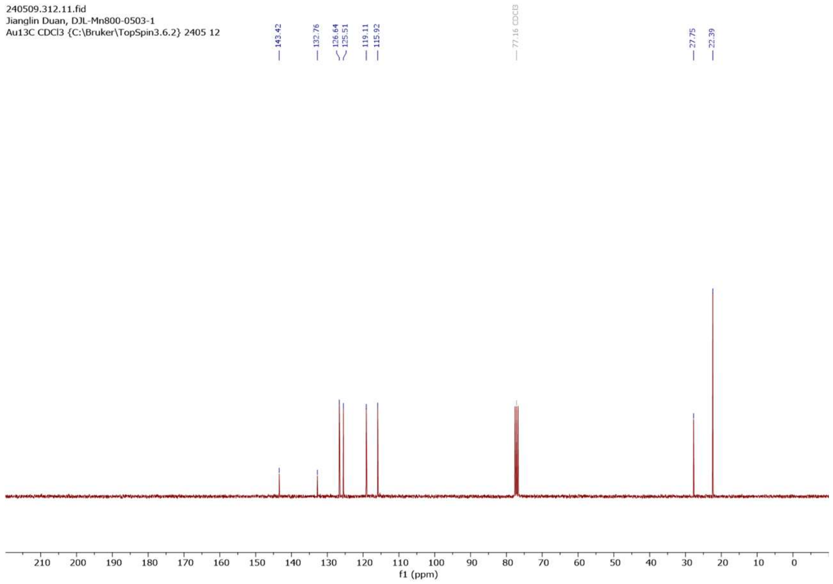

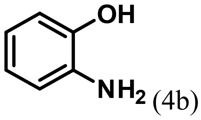

(4b)

1H NMR (300 MHz, DMSO) δ 8.89 (s, br, 1H), 6.61 (dd, J = 7.7, 1.4, 0.4 Hz, 1H), 6.58 – 6.48 (m, 2H), 6.37 (ddd, J = 7.7, 7.0, 2.0 Hz, 1H), 4.43 (s, 2H).

13C NMR (75 MHz, DMSO) δ 143.96, 136.53, 119.48, 116.38, 114.40, 114.35.

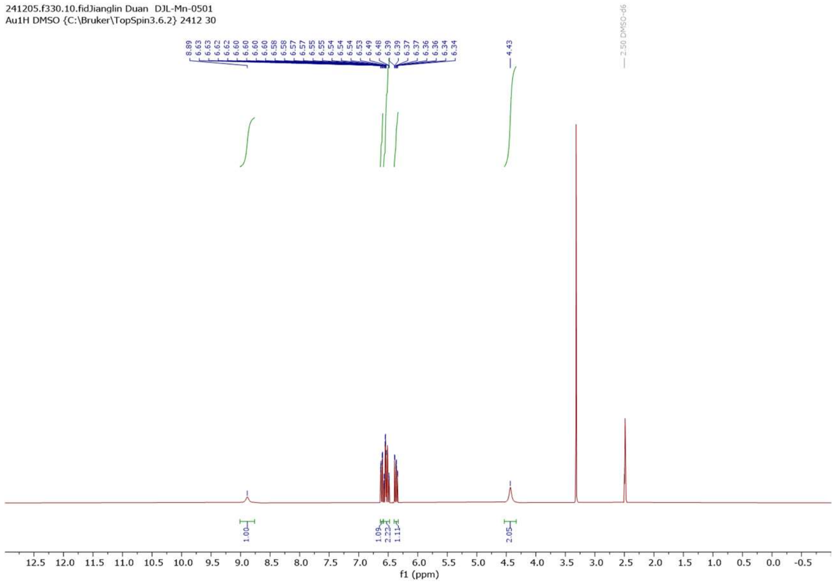

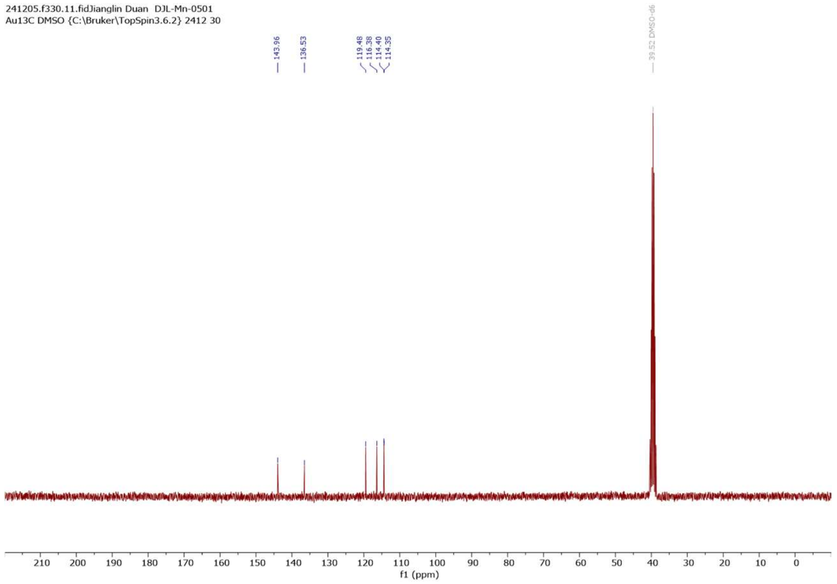

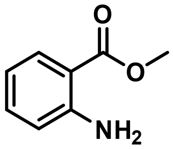

(5b)

1H NMR (300 MHz, $CDCl_3$) δ 7.89 – 7.83 (m, 1H), 7.24 (d, J = 1.6 Hz, 1H), 6.68 – 6.60 (m, 2H), 5.72 (s, 2H), 3.87 (s, 3H).

13C NMR (75 MHz, $CDCl_3$) δ 168.69, 150.55, 134.19, 131.32, 116.77, 116.37, 110.86, 51.61.

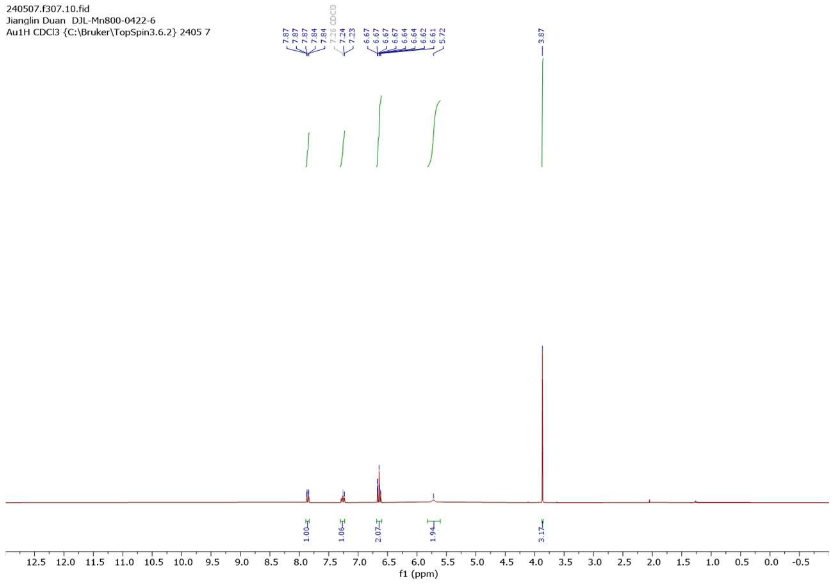

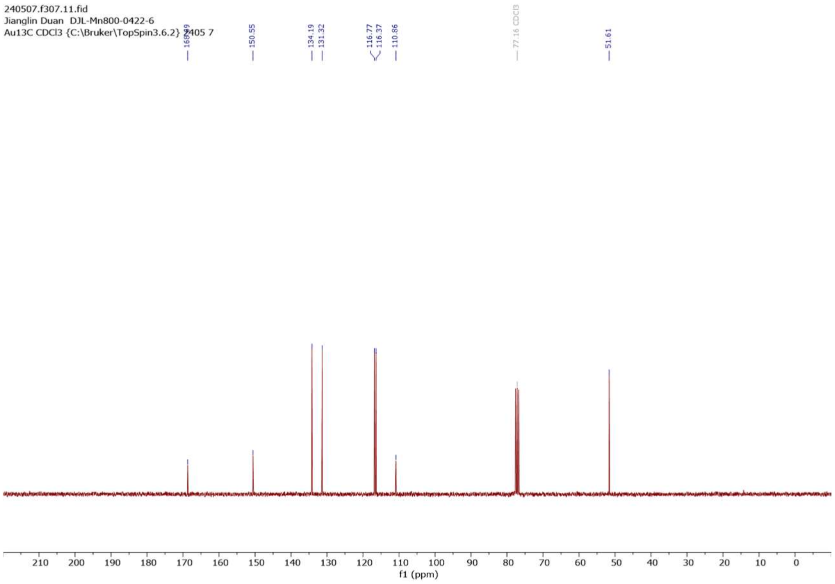

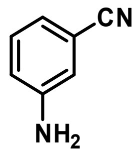

(6b)

1H NMR (300 MHz, $CDCl_3$) δ 7.20 (t, J = 8.1, 0.6 Hz, 1H), 7.01 (d, J = 7.6, 1.5, 1.0 Hz, 1H), 6.93 – 6.82 (m, 2H), 3.87 (s, 2H).

13C NMR (75 MHz, $CDCl_3$) δ 147.03, 130.19, 122.15, 119.31, 117.58, 113.09.

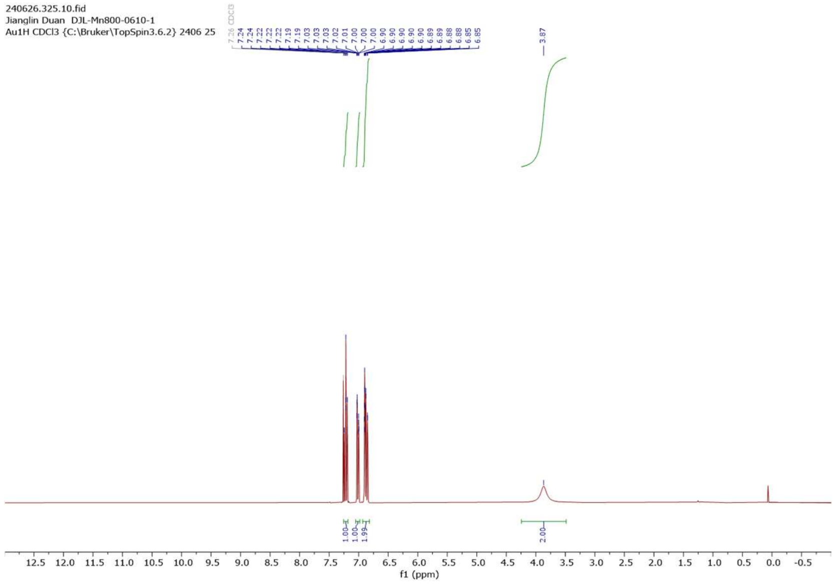

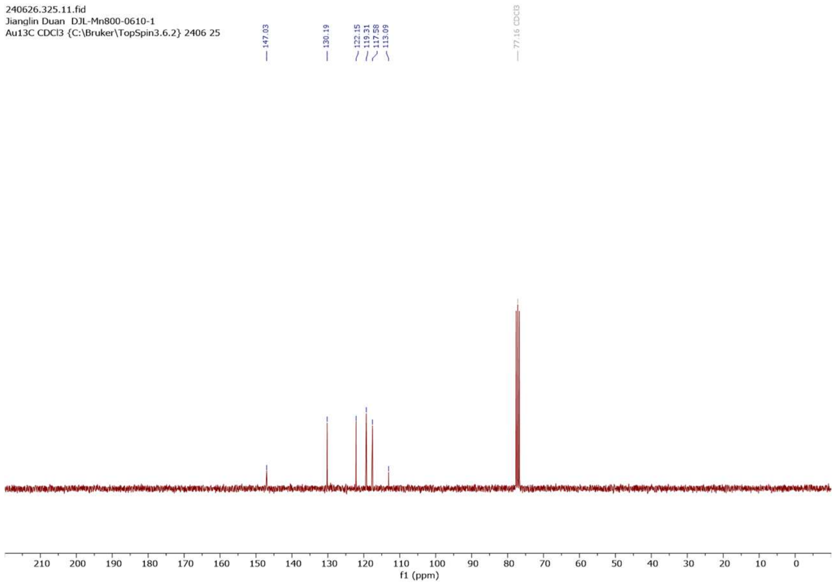

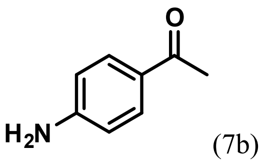

(7b)

1H NMR (300 MHz, $CDCl_3$) δ 7.80 (d, J = 8.7 Hz, 2H), 6.64 (d, J = 8.8 Hz, 2H), 4.14 (s, 2H), 2.50 (s, 3H).

13C NMR (75 MHz, $CDCl_3$) δ 196.61, 151.23, 130.93, 128.04, 113.85, 26.21.

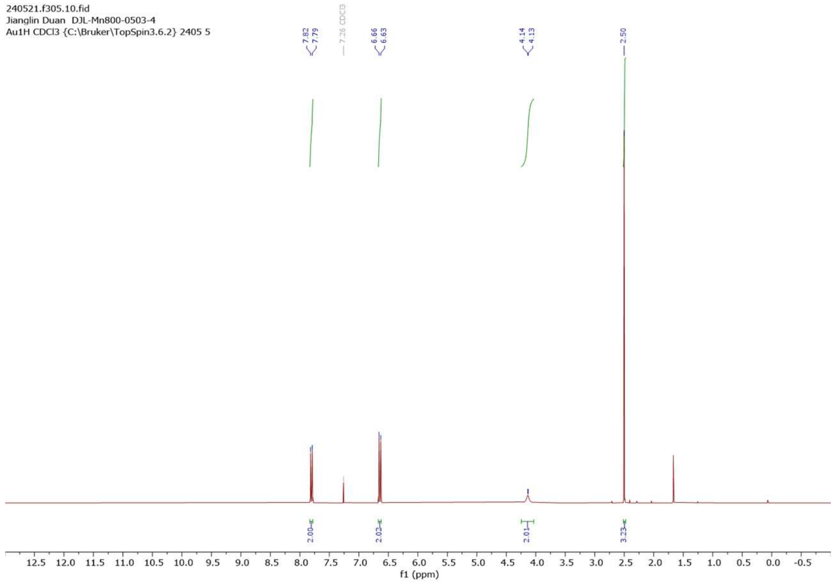

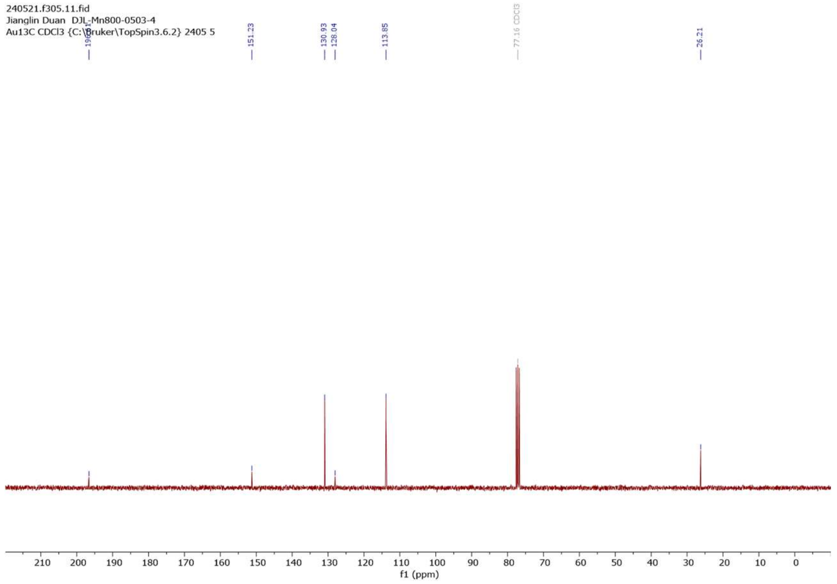

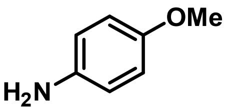

(8b)

1H NMR (400 MHz, $CDCl_3$) δ 6.75 (d, J = 8.9 Hz, 2H), 6.65 (d, J = 9.0 Hz, 2H), 3.75 (s, 3H), 3.42 (s, 2H).

13C NMR (101 MHz, $CDCl_3$) δ 152.93, 140.05, 116.54, 114.93, 55.86.

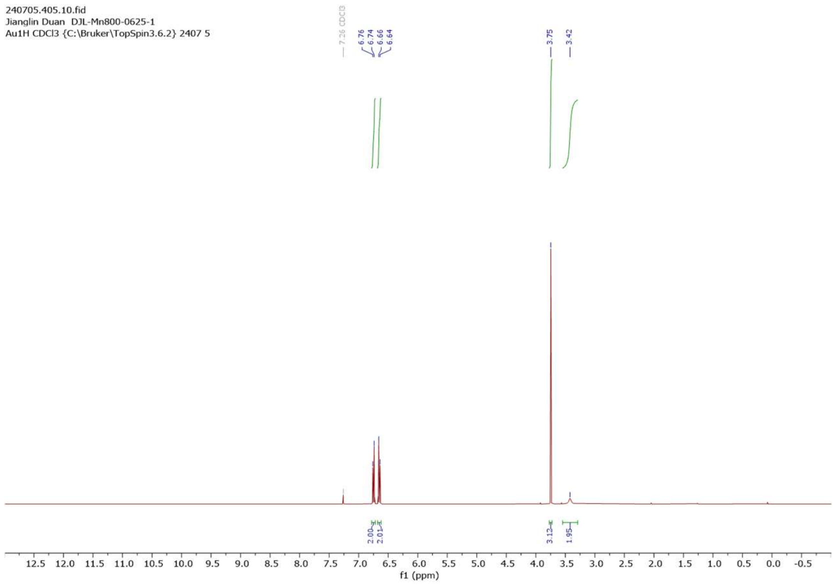

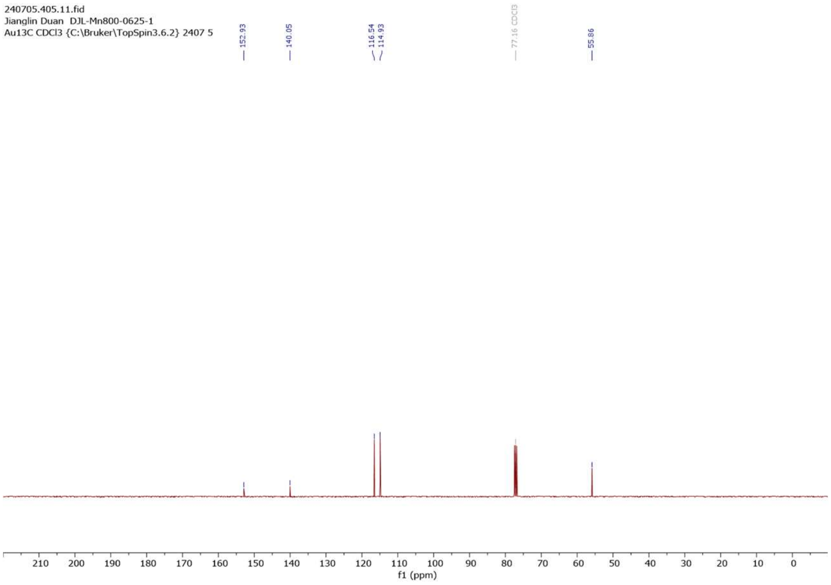

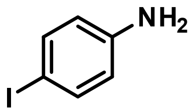

(9b)

1H NMR (300 MHz, $CDCl_3$) δ 7.41 (d, J = 8.8 Hz, 1H), 6.47 (d, J = 8.8 Hz, 1H), 3.67 (s, 1H).

13C NMR (75 MHz, $CDCl_3$) δ 146.11, 137.97, 117.34, 79.42.

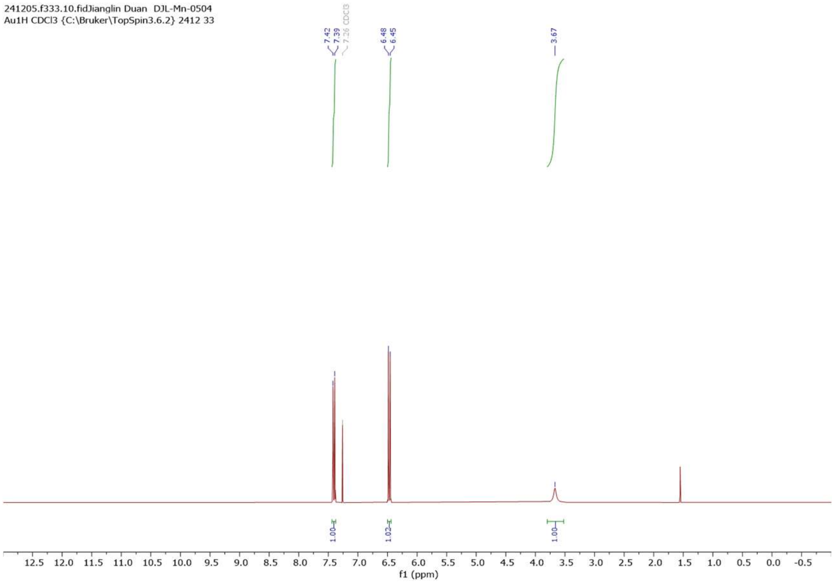

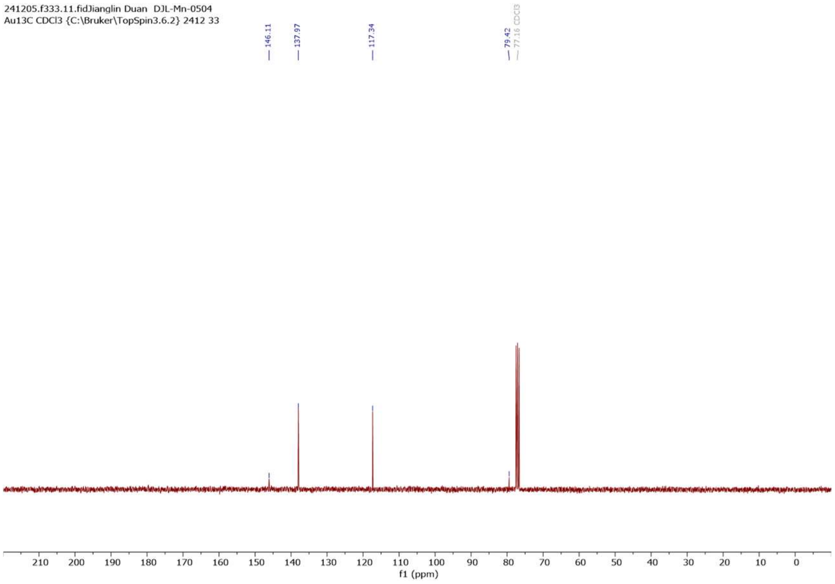

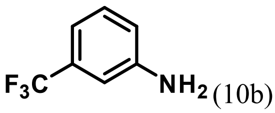
(10b)

1H NMR (300 MHz, $CDCl_3$) δ 7.30 – 7.21 (t, 1H), 7.05 – 6.97 (m, 1H), 6.93 – 6.88 (m, 1H), 6.82 (ddt, J = 8.0, 1.6, 0.8 Hz, 1H), 3.83 (s, 2H).
13C NMR (75 MHz, $CDCl_3$) δ 146.86, 131.91(q), 129.86, 126.14(q), 118.11, 115.11 (q), 111.43 (q).
19F NMR (282 MHz, $CDCl_3$) δ -62.89.

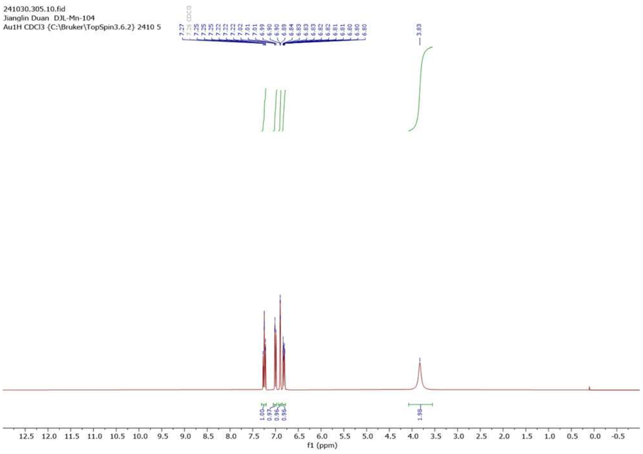

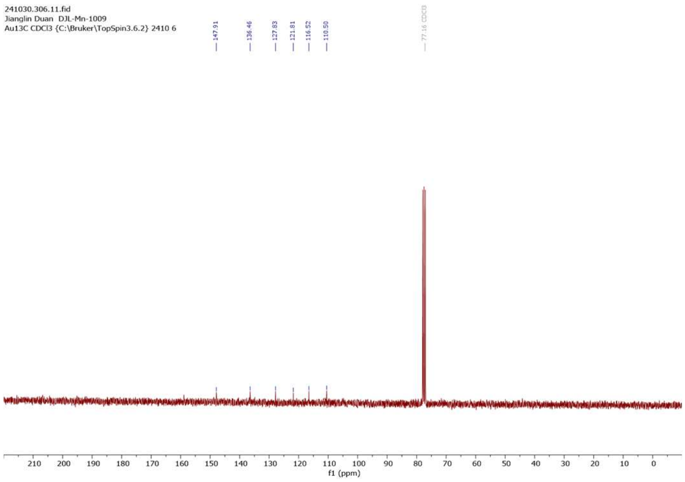

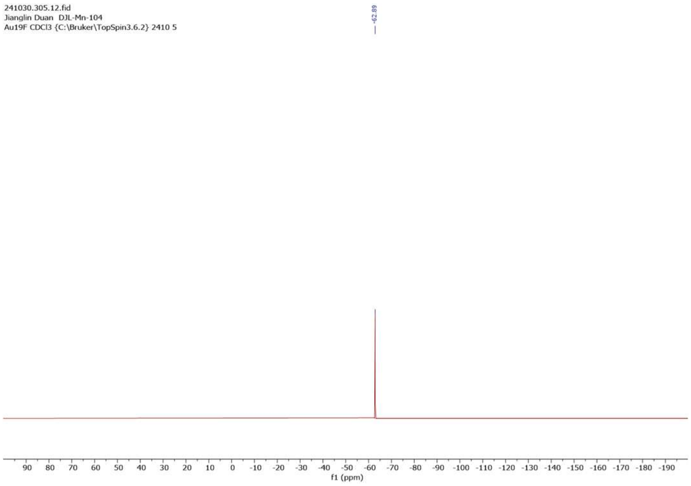

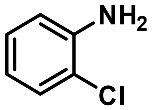 (11b)

1H NMR (300 MHz, $CDCl_3$) δ 7.28 (d, J = 8.0 Hz, 1H), 7.09 (t, J = 7.7 Hz, 1H), 6.77 (d, J = 8.0 Hz, 1H), 6.72 (t, J = 7.7 Hz, 1H), 4.03 (s, 2H)

13C NMR (75 MHz, $CDCl_3$) δ 143.02, 129.53, 127.74, 119.40, 119.14, 115.98.

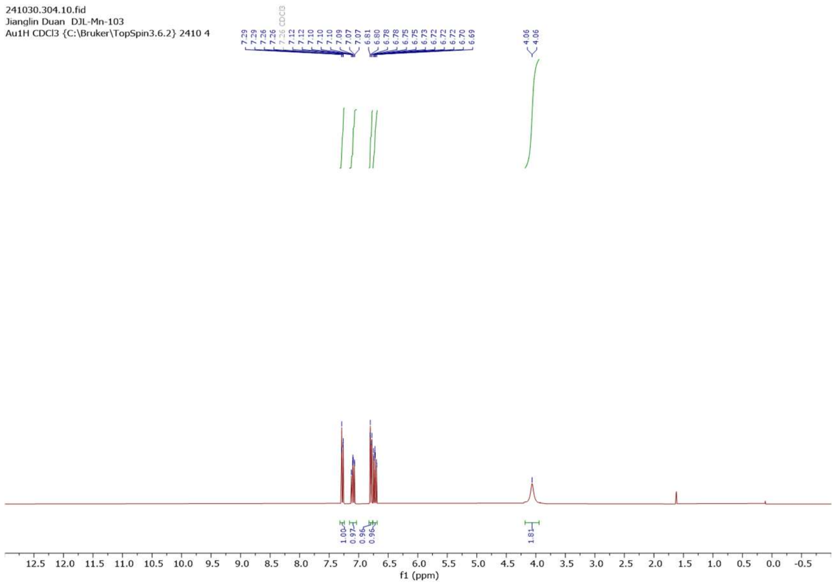

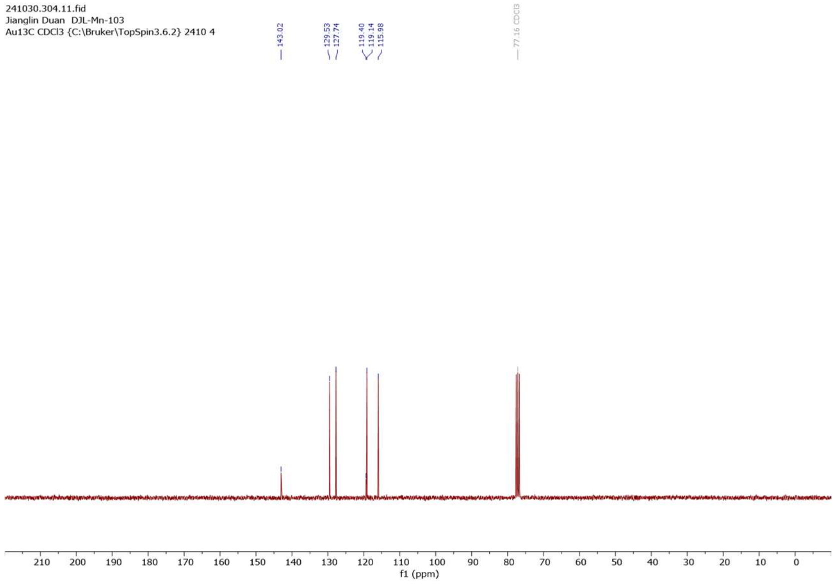

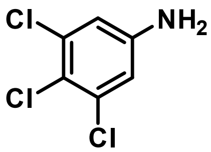

(12b)

1H NMR (300 MHz, $CDCl_3$) δ 6.70 (s, 2H), 3.77 (s, 2H).

13C NMR (75 MHz, $CDCl_3$) δ 145.72, 134.43, 115.19.

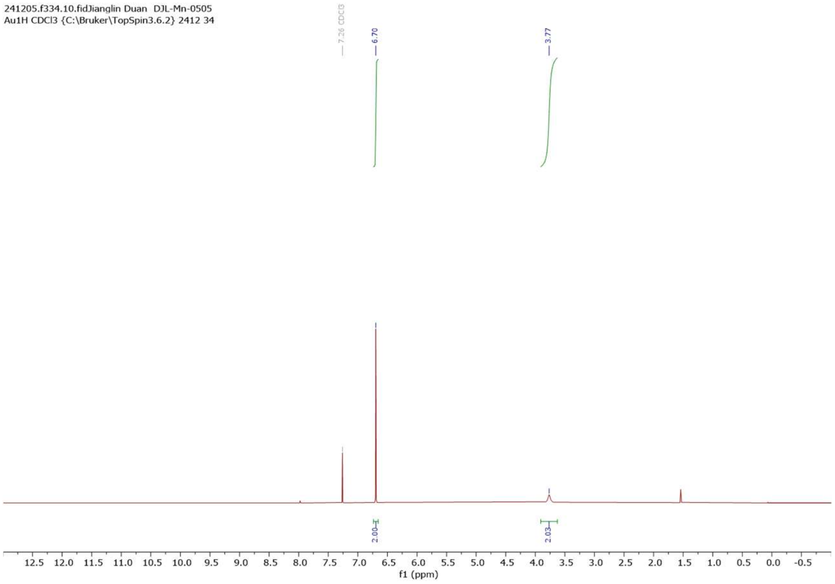

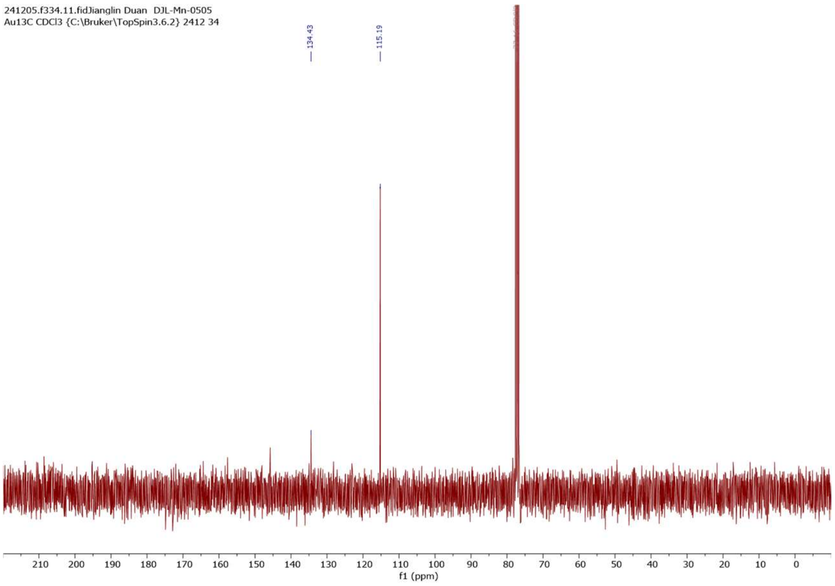

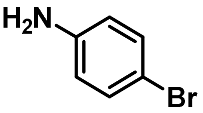
(13b)

1H NMR (300 MHz, CDCl3): δ 7.23 (d, 2H, J = 8.4 Hz), 6.55 (d, 2H, J = 8.4 Hz), 3.66 (br s, 2H); 13C NMR (75 MHz, CDCl3): δ 145.6k, 132.2, 116.8, 110.4.

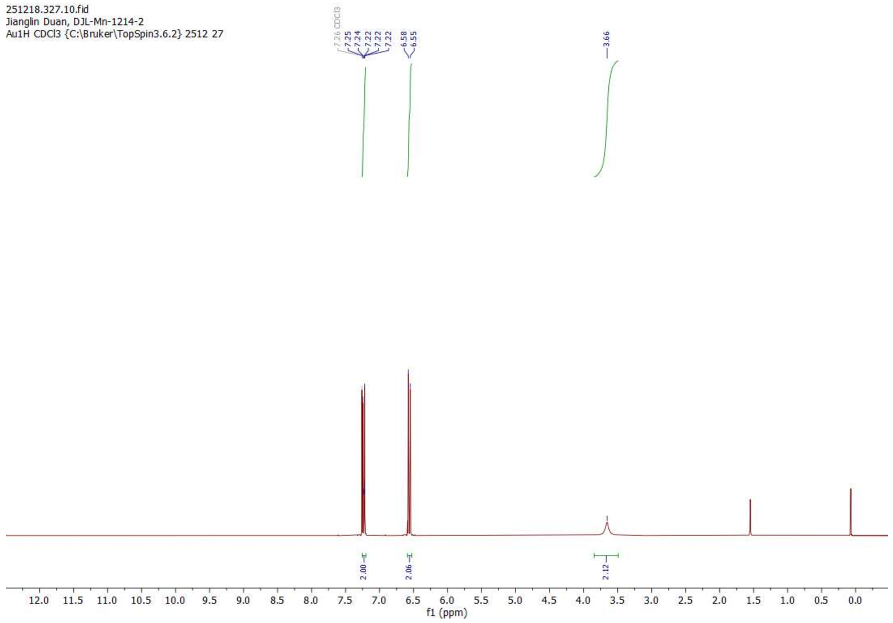

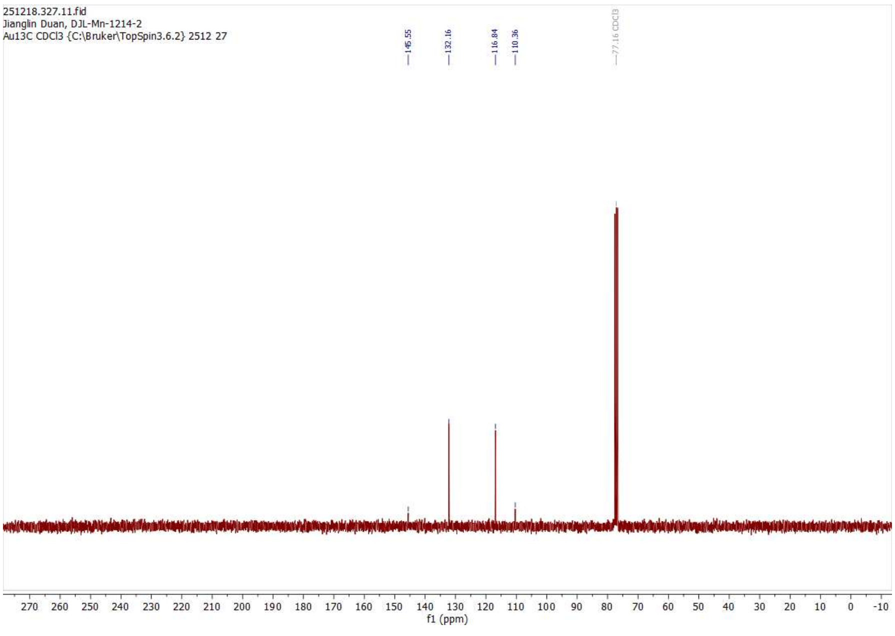

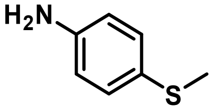

(14b)

1H NMR (300 MHz, $CDCl_3$) δ 7.18 (d, J = 8.7 Hz, 2H), 6.63 (d, J = 8.7 Hz, 2H), 3.63 (s, 2H), 2.41 (s, 3H).

13C NMR (75 MHz, $CDCl_3$) δ 145.22, 131.22, 125.97, 115.88, 18.94.

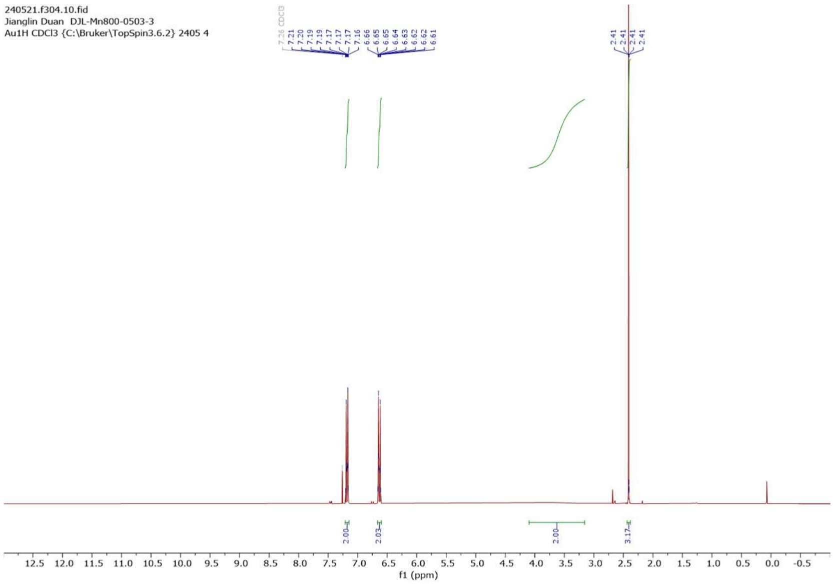

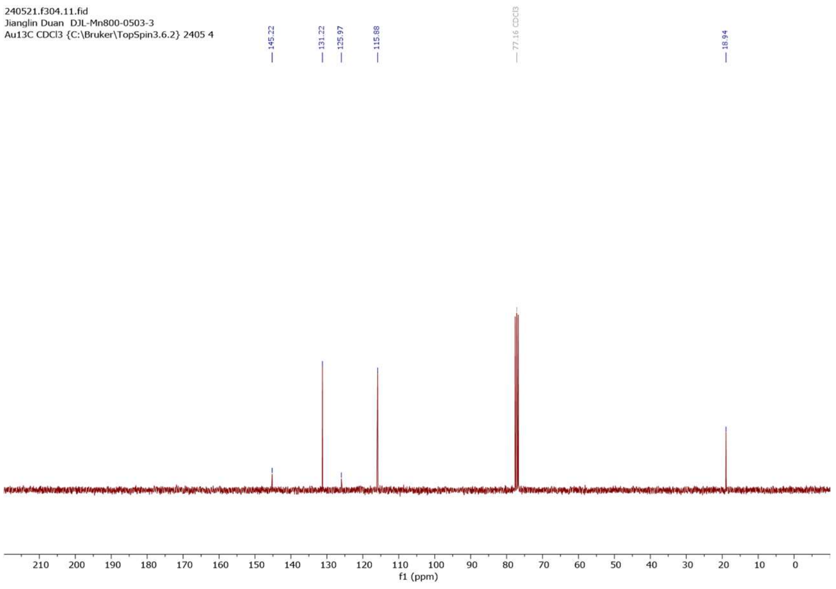

1

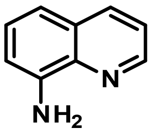

(15b)

1H NMR (300 MHz, $CDCl_3$) δ 8.76 (dd, J = 4.2, 1.7 Hz, 1H), 8.07 (dd, J = 8.3, 1.7 Hz, 1H), 7.40 – 7.30 (m, 2H), 7.15 (dd, J = 8.2, 1.2 Hz, 1H), 6.93 (dd, J = 7.5, 1.3 Hz, 1H), 4.98 (s, br, 2H).

13C NMR (75 MHz, $CDCl_3$) δ 147.91, 136.46, 127.83, 121.81, 116.52, 110.50.

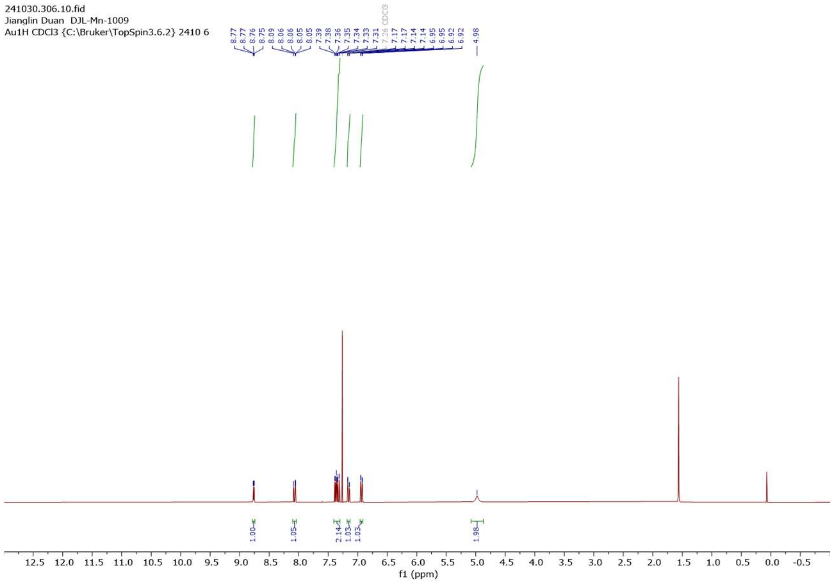

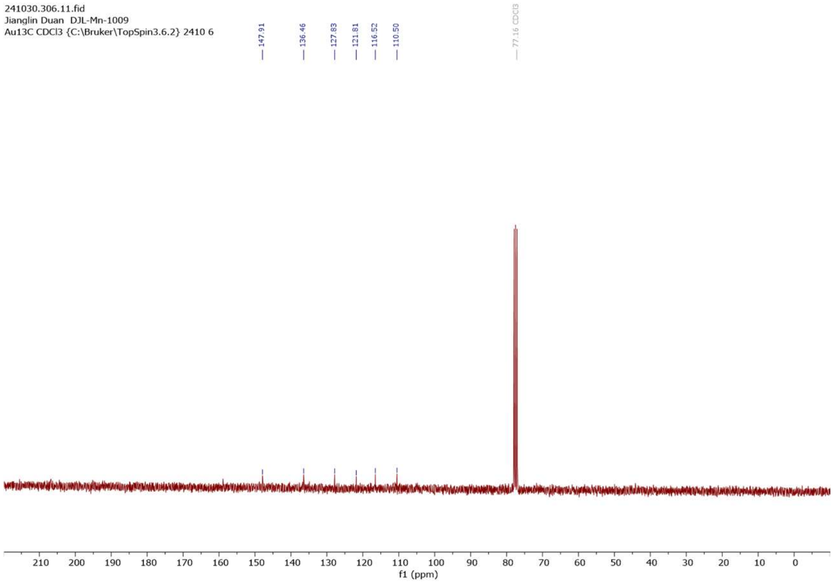

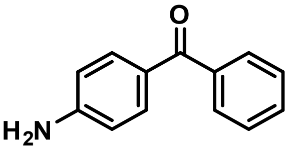

(16b)

1H NMR (300 MHz, $CDCl_3$) δ 7.76 – 7.69 (m, 4H), 7.57 – 7.50 (m, 1H), 7.49 – 7.42 (m, 2H), 6.71 – 6.64 (m, 2H), 4.14 (s, 2H).

13C NMR (75 MHz, $CDCl_3$) δ 151.02, 133.09, 131.55, 129.68, 128.22, 113.79.

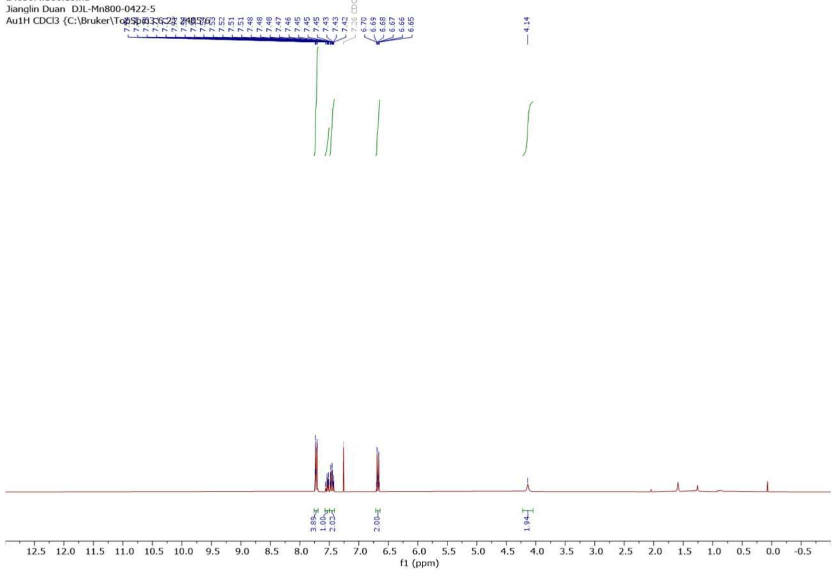

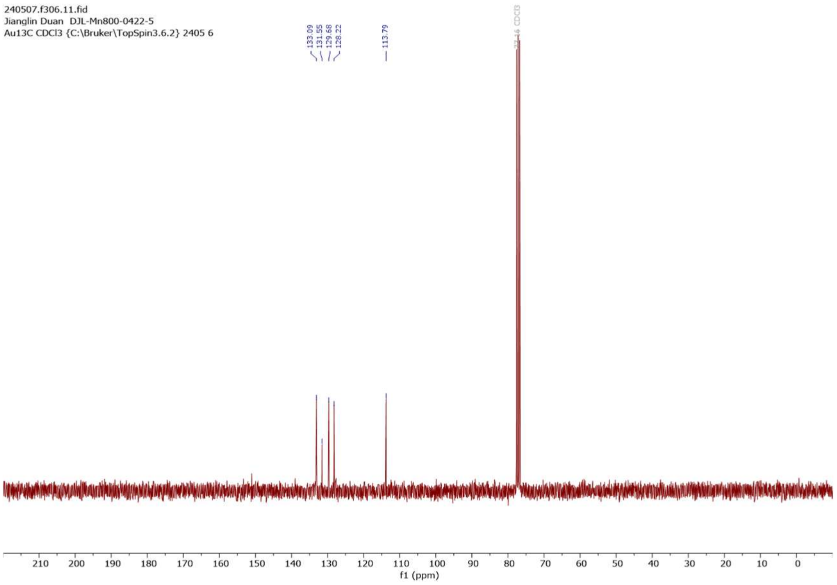

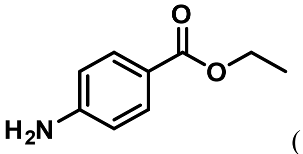

(17b)

1H NMR (300 MHz, $CDCl_3$) δ 7.86 (d, J = 8.7 Hz, 2H), 6.64 (d, J = 8.8 Hz, 2H), 4.32 (q, J = 7.1 Hz, 2H), 4.04 (s, 2H), 1.36 (t, J = 7.1 Hz, 3H).

13C NMR (75 MHz, $CDCl_3$) δ 166.83, 150.83, 131.69, 120.30, 113.92, 60.44, 14.57.

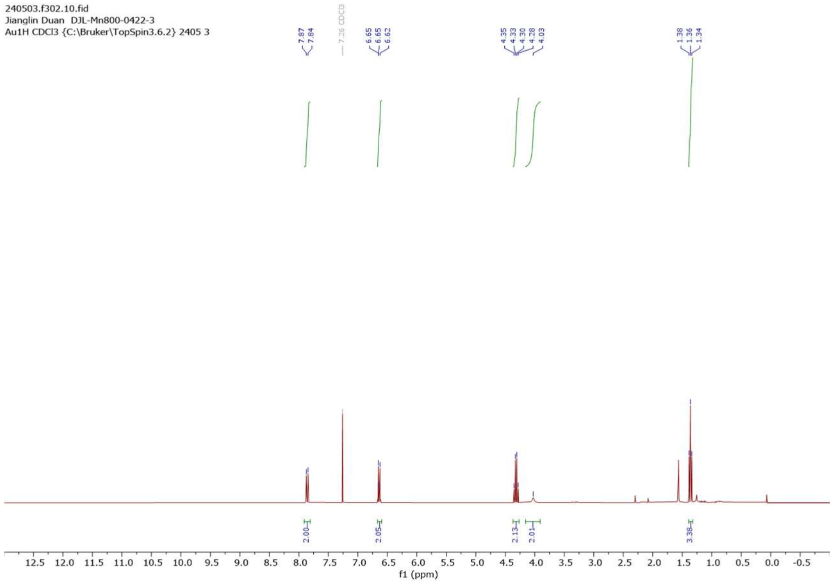

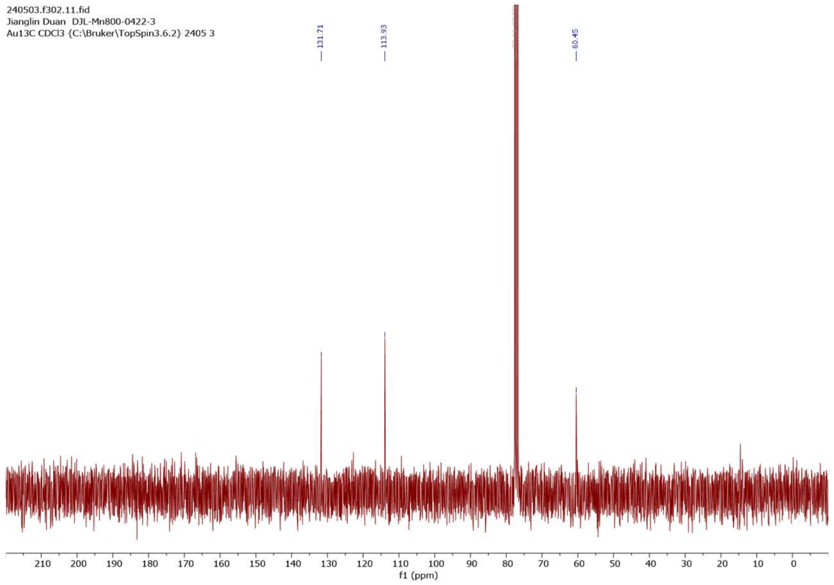

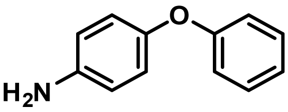

(18b)

1H NMR (400 MHz, $CDCl_3$) δ 7.27 – 7.21 (m, 2H), 7.02 – 6.94 (m, 1H), 6.92 – 6.87 (m, 2H), 6.86 – 6.81 (m, 2H), 6.68 – 6.61 (m, 2H), 3.54 (s, 2H).

13C NMR (101 MHz, $CDCl_3$) δ 159.03, 148.74, 142.81, 129.66, 122.19, 121.28, 117.35, 116.38.

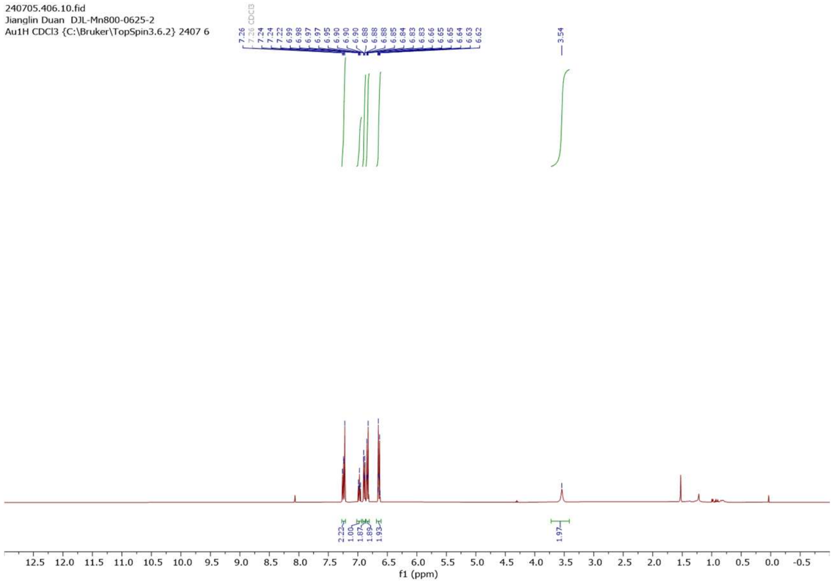

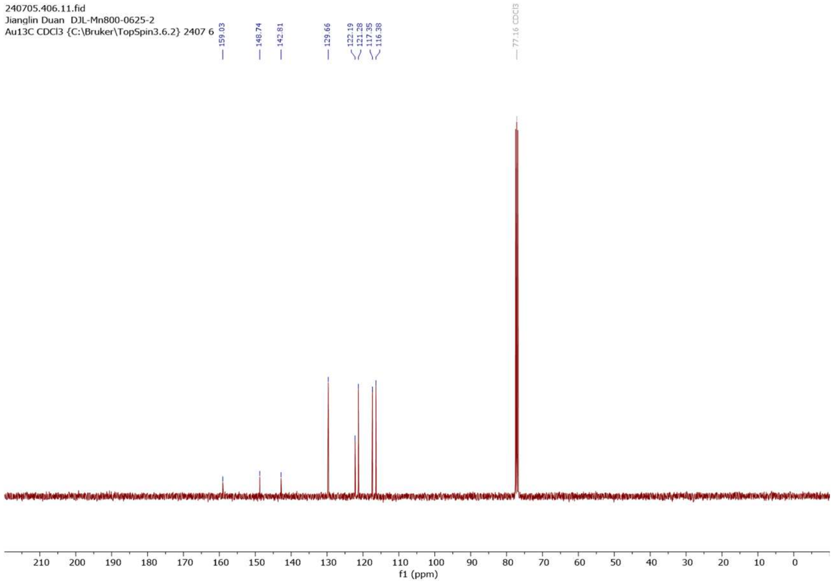

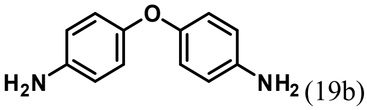
(19b)

1H NMR (300 MHz, $CDCl_3$) δ 6.80 (d, J = 8.8 Hz, 1H), 6.64 (d, J = 9.0 Hz, 1H).

13C NMR (75 MHz, $CDCl_3$) δ 150.90, 119.68, 116.36.

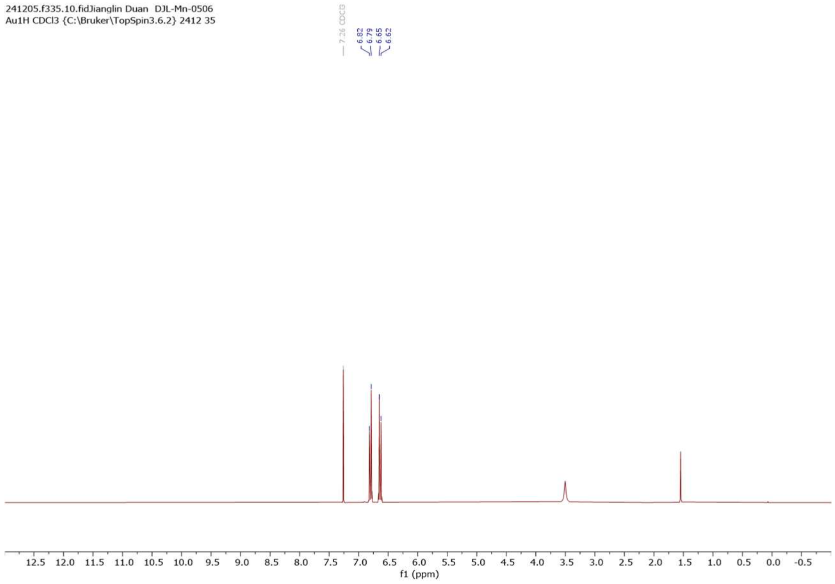

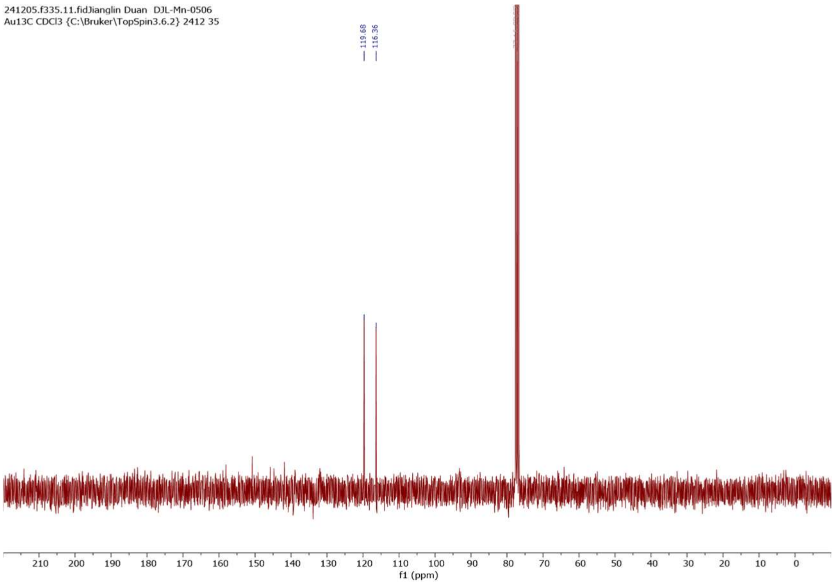

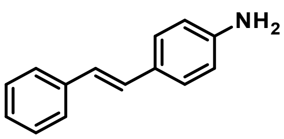

(20b)

1H NMR (300 MHz, $CDCl_3$) δ 7.51 – 7.45 (d, 2H), 7.38 – 7.31 (dt, 4H), 7.25 – 7.19 (m, 1H), 7.04 (d, J = 16.3 Hz, 1H), 6.93 (d, J = 16.3 Hz, 1H), 6.68 (d, J = 8.5 Hz, 2H), 3.74 (s, 2H).

13C NMR (75 MHz, $CDCl_3$) δ 146.28, 138.09, 128.82, 128.73, 128.17, 127.89, 127.03, 126.24, 125.25, 115.33.

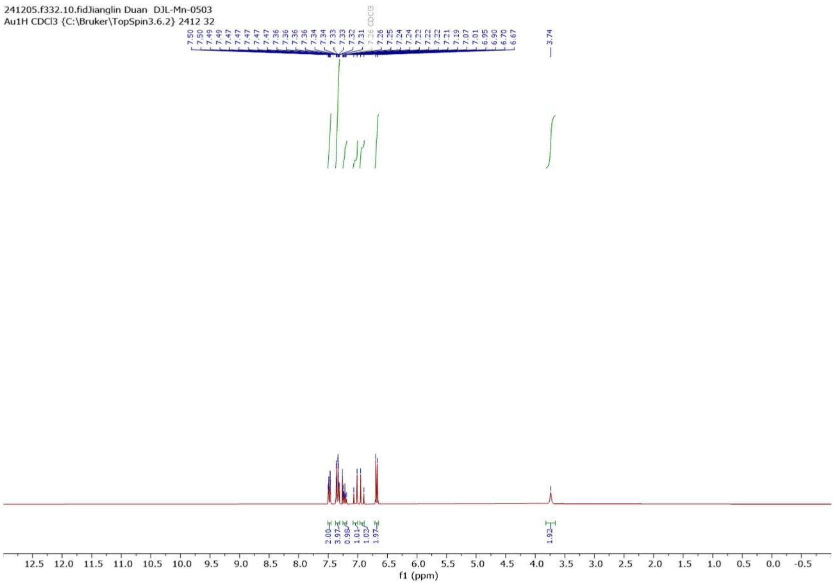

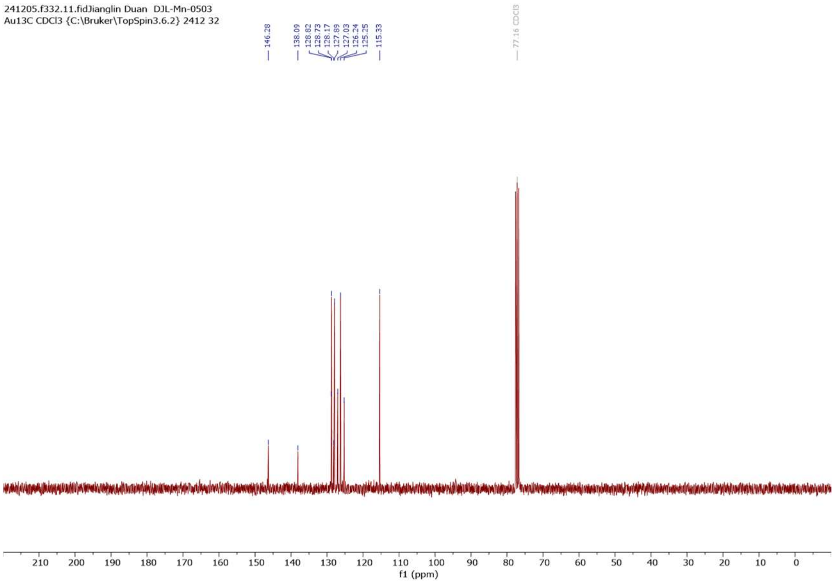

4 References:

(1) Luk, J.; Oates, C.; Garcia, J.; Clarke, M.; Kumar, A. Manganese-Catalyzed Hydrogenation of Amides and Polyurethanes: Is Catalyst Inhibition an Additional Barrier to the Efficient Hydrogenation of Amides and Their Derivatives? *Organometallics* **2024**, *43*(2), 85–93.

(2) Zubar, V.; Dewanji, A.; Rueping, M. Chemoselective Hydrogenation of Nitroarenes Using an Air-Stable Base-Metal Catalyst. *Org. Lett.* **2021**, *23*, 2742– 2747.

(3) Weber, S.; Brünig, J.;Veiros, L. F.; Kirchner, K. Manganese-Catalyzed Hydrogenation of Ketones under Mild and Base-free Conditions. *Organometallics* **2021**, *40*, 1388– 1394.

(4) Zubar, V.; Lichtenberger, N.; Schelwies, M.; Oeser, T.; Hashmi, A. S. K.; Schaub, T. Manganese-Catalyzed Hydrogenation of Sclareolide to Ambradiol. *ChemCatChem* **2021**, *14* (1), e202101443.

(5) Kaithal, A.; Hölscher, M.; Leitner, W. Catalytic Hydrogenation of Cyclic Carbonates using Manganese Complexes. *Angew. Chem., Int. Ed.* **2018**, *57*, 13449–13453.

(6) Yang, F.; Wang, M.; Liu, W.; Yang, B.; Wang, Y.; Luo, J.; Tang, Y.; Hou, L.; Li, Y.; Li, Z.; Zhang, B.; Yang, W.; Li, Y. Atomically Dispersed Ni as the Active Site towards Selective Hydrogenation of Nitroarenes. *Green Chem.* **2019**, *21*, 704–711.

(7) Zhou, D.; Zhang, L.; Liu, X.; Qi, H.; Liu, Q.; Yang, J.; Su, Y.; Ma, J.; Yin, J.; Wang, A. Tuning the Coordination Environment of Single-Atom Catalyst M–N–C towards Selective Hydrogenation of Functionalized Nitroarenes. *Nano Res.* **2022**, *15*, 519.

(8) Qu, Y.; Yang, H.; Wang, S.; Chen, T.; Wang, G. Hydrogenation of Nitrobenzene to Aniline Catalyzed by C60-Stabilized Ni. *Catal. Commun.* **2017**, *97*, 83–87.

(9) Gao, R.; Pan, L.; Wang, H.; Yao, Y.; Zhang, X.; Wang, L.; Zou, J. Breaking Trade-Off between Selectivity and Activity of Nickel-Based Hydrogenation Catalysts by Tuning Both Steric Effect and d-Band Center. *Adv. Sci.* **2019**, *6* (10), 1900054.

(10) Wei, X.; Zhou, M.; Zhang, X.; Wang, X.; Wu, Z. Amphiphilic mesoporous sandwich-structured catalysts for selective hydrogenation of 4-Nitrostyrene in water. *ACS Appl. Mater. Interfaces* **2019**, *11*, 39116–39124.

(11) Li, M.; Chen, S.; Jiang, Q.; Chen, Q.; Wang, X.; Yan, Y.; Liu, J.; Lv, C.; Ding, W.; Guo, X. Origin of the Activity of Co–N–C Catalysts for Chemoselective Hydrogenation of Nitroarenes. *ACS Catal.* **2021**, *11*, 3026–3039.

(12) Sun, X.; Olivos-Suarez, A.; Osadchii, D.; Romero, M.; Kapteijn, F.; Gascon, J. Show more Single cobalt sites in mesoporous N-doped carbon matrix for selective catalytic hydrogenation of nitroarenes. *J. Catal.* **2018**, *357*, 20-28.

(13) Jagadeesh, R. V.; Stemmler, T.; Surkus, A.-E.; Bauer, M.; Pohl, M.-M.; Radnik, J.; Junge, K.; Junge, H.; Brückner, A.; Beller, M. Nat. Protoc. **2015**, 10, 916–926.

(14) Xu, S.; Yu, D.; Liao, S.; Ye, T.; Sheng, H. Nitrogen-doped carbon supported iron oxide as efficient catalysts for chemoselective hydrogenation of nitroarenes. *RSC Adv.* **2016**, *6*, 96431–96435.
